# Comparative Evaluation of Deep Object Detection Architectures for Automatic Target Recognition in Real-World AUV-Acquired Side-Scan Sonar Imagery

**DOI:** 10.3390/s26206486

**Published:** 2026-10-13

**Authors:** Daniel Powarzyński, Norbert Sigiel, Aleksander Cirocki, Mateusz Toporek

**Affiliations:** 1 Faculty of Mechanical and Electrical Engineering, Polish Naval Academy, 81-127 Gdynia, Poland; 26207@edu.amw.gdynia.pl (A.C.); 26195@edu.amw.gdynia.pl (M.T.); 2 Faculty of Navigation and Naval Weapons, Polish Naval Academy, 81-127 Gdynia, Poland; n.sigiel@amw.gdynia.pl

**Keywords:** automatic target recognition (ATR), side-scan sonar (SSS), autonomous underwater vehicles (AUVs), mine-like object detection, transfer learning

## Abstract

Reliable detection of underwater objects is important for mine countermeasures, unexploded-ordnance surveys, navigational-hazard assessment, and subsea infrastructure inspection. Interpretation of side-scan sonar (SSS) imagery is still largely performed by trained human operators, making post-mission analysis time-consuming when large volumes of data are collected. Automatic Target Recognition (ATR) can support this process by automatically detecting potentially relevant contacts and reducing the amount of imagery requiring detailed manual inspection. However, the ability of deep-learning detectors to generalize across real underwater missions remains insufficiently investigated. This study presents a comparative evaluation of five deep object-detector configurations, Faster R-CNN, RetinaNet, SSD, YOLOX, and DETR, for ATR using real-world SSS imagery acquired by autonomous underwater vehicles (AUVs). The development dataset comprised 12,066 image patches containing 18,253 annotated instances from three classes, acquired during five measurement missions. Two additional complete missions were excluded from all stages of model development and used as independent cross-mission tests of the same physical reference objects under previously unseen acoustic and acquisition conditions. Within-development validation ranked Faster R-CNN first, with a three-class ${\mathrm{mAP}}_{50:95}$ of 0.451 and a recalculated two-class value of 0.568; this validation ranking was used for model selection and was not treated as evidence of independent generalization. On the held-out missions, SSD achieved the highest primary ${\mathrm{mAP}}_{50:95}$ on Test A (0.614), whereas YOLOX achieved the highest value on Test B (0.084) and the highest inference throughput. A retrospective comparison of the validation-selected and terminal training checkpoints showed greater checkpoint sensitivity on Test A than on Test B, although SSD and YOLOX remained the highest-performing configurations on their respective missions. The substantial variation in detector ordering and performance between the held-out missions highlights the influence of mission-specific conditions on ATR performance and demonstrates the importance of evaluating sonar detectors on complete real-world missions rather than relying on within-development validation rankings as evidence of generalization.

## 1. Introduction

Reliable detection, localization, and classification of objects resting on the seafloor are essential to maritime situational awareness and decision-making in both military and civilian operations. Side-scan sonar (SSS) is widely used in mine countermeasures, unexploded ordnance surveys, navigational-hazard assessment, hydrographic and geological mapping, marine archaeology, environmental monitoring, and the inspection of subsea infrastructure. When integrated with an autonomous underwater vehicle (AUV), SSS enables low-altitude seabed imaging along repeatable survey lines while reducing the need to expose personnel or surface platforms directly to potentially hazardous areas [1,2,3]. However, systematic surveys may generate extensive volumes of imagery whose exhaustive manual post-mission analysis is time-consuming and strongly dependent on operator experience. This creates a need for automatic target recognition (ATR) methods capable of supporting contact detection, classification, and prioritization [2,3,4].

A side-scan sonar does not produce an optical photograph of the seabed. Its transducers emit acoustic pulses laterally to the port and starboard sides of the carrier platform, and the returning backscattered signal is recorded after interaction with the water column, seabed, and insonified objects. The two-way acoustic travel time is converted into slant range, whereas the amplitude of the received signal is represented as image intensity. Successive acoustic pings acquired along the vehicle trajectory form a two-dimensional sonar record, commonly visualized as a waterfall image [1]. An object protruding above the seabed typically produces a relatively bright acoustic highlight followed by a darker acoustic shadow corresponding to a portion of the bottom that was not directly insonified. The shadow is therefore an acoustic consequence of geometric occlusion rather than an optical representation of the physical object. Its length, continuity, and morphology provide information about target height, orientation, and acquisition geometry [1,5].

The appearance of a sonar contact is conditional on the measurement process and cannot be treated as an invariant representation of a physical object. Sonar bandwidth, nominal operating frequency, sensor altitude, slant range, grazing angle, platform motion, target aspect, degree of burial, surface material, acoustic-impedance contrast, seabed composition and roughness, ambient and instrumental noise, and reverberation can all modify the recorded highlight–shadow pattern [1,2,5]. Consequently, repeated observations of the same target may differ considerably, whereas rocks, sedimentary structures, anthropogenic debris, and other benign contacts may resemble mine-like objects under particular insonification conditions. Reliable interpretation therefore requires joint assessment of complementary cues, including echo response or target-to-background contrast, apparent dimensions, geometric shape, acoustic-shadow characteristics, and local environmental context [2,5,6]. The problem is particularly demanding for compact, low-profile, partially buried, or unfavorably oriented targets whose acoustic signatures may occupy only a small image area or merge with heterogeneous seabed clutter [2,5].

Classical sonar ATR systems generally employ a sequential processing chain comprising geometric correction, candidate detection, segmentation of acoustic highlights and shadows, handcrafted feature extraction, and final classification. Representative methods include automatic extraction of highlight and shadow features from mine-like contacts [7], texture descriptors combined with active contours [8], template matching between measured and model-generated sonar signatures [9], and explicit use of environmental information to adapt the classification process [6]. Such methods provide physically interpretable intermediate results, which is particularly valuable in safety-critical applications. Their performance may nevertheless be limited by error propagation between consecutive stages and by the sensitivity of handcrafted descriptors to changes in sensor configuration, seabed type, target aspect, and acquisition geometry. Reviews of sonar ATR therefore identify robustness across environments, sensors, and datasets as a persistent challenge [2,10].

Deep learning shifted the emphasis from manually specified descriptors toward hierarchical feature representations learned directly from data. Early studies demonstrated that convolutional neural networks could extract discriminative representations from sonar imagery, including target classification in synthetic aperture sonar images [11] and target recognition in SSS imagery [12]. Transfer learning and semi-synthetic sample generation were subsequently investigated to alleviate the limited availability of annotated sonar data [13], while comparative studies examined how optical-image backbones and pretrained weights perform after adaptation to the SSS domain [14]. These developments improved feature-learning capability but also highlighted two important methodological distinctions.

First, image classification and object detection are not equivalent tasks. A classifier assigns a label to a predefined image patch, whereas an object detector must localize and classify one or more contacts in the presence of extensive seabed background. Classification accuracy obtained from manually cropped and often balanced contact images therefore cannot be compared directly with average precision obtained from an object detection task. Second, initialization with weights learned from optical images does not eliminate the acoustic-to-optical domain gap. Sonar imagery contains direction-dependent highlights and shadows, range-dependent intensity variation, reverberation, speckle, and mission-specific acquisition-geometry effects that are not represented in conventional optical-image datasets [10,13,14].

Recent research has increasingly adopted end-to-end object detectors and focused on small-target sensitivity, multi-scale feature representation, inference speed, and potential deployment on resource-constrained maritime platforms. Steiniger et al. compared YOLOv2, YOLOv3, and a compact convolutional detector on SSS data [15]. A transformer-enhanced YOLOv5 architecture was proposed for real-time maritime-object detection [16], while an AUV-oriented processing chain was developed for real-time target detection from SSS navigation strips [17]. Lightweight architectures have also been designed specifically for SSS applications [18]. More recently, Liu et al. compared four contemporary YOLO variants and analyzed the influence of transfer learning [19], whereas Xie et al. introduced a target-presence-aware coarse-to-fine framework intended to reduce unnecessary inference over target-free areas [20]. Collectively, these studies demonstrate rapid progress; however, a substantial part of the recent comparative evidence remains concentrated within the YOLO family or around individual task-specific modifications.

Direct comparison of published results remains difficult because studies differ in sonar modality, sensor configuration, target definitions, numbers of classes, image dimensions, annotation policies, use of synthetic data, data-partitioning strategies, and evaluation metrics. Random image-level partitions may also provide a less demanding assessment of generalization than testing on complete survey missions excluded from model development. Existing comparative studies have primarily considered alternative CNN backbones [14], a limited set of earlier detectors [15], or successive variants of the same detector family [19]. Reviews have therefore emphasized the need for representative real-world datasets, common evaluation criteria, and experimental protocols reflecting the AUV–SSS processing chain [3,10,21]. Within the literature examined in this study, systematic evaluations of substantially different detector paradigms using a common real-world AUV-acquired development dataset and complete held-out survey missions remain insufficiently documented.

To address this gap, the present study evaluates five complete object-detector configurations based on representative deep detection architectures: Faster R-CNN, a two-stage detector based on region proposals [22]; RetinaNet, a dense one-stage detector employing focal loss [23]; SSD, a computationally efficient one-stage detector using predictions from multiple feature-map scales [24]; YOLOX, an anchor-free detector with a decoupled head and dynamic label assignment [25]; and DETR, which formulates detection as end-to-end set prediction using a transformer encoder–decoder [26]. The configurations were selected to represent different detection paradigms rather than incremental variants of a single model family.

All configurations were evaluated using the same sonar-domain development dataset, a fixed training–validation partition, a common maximum training budget, the same effective batch size, validation-based checkpoint selection, and consistent evaluation criteria. Architecture-specific input resolutions, data-augmentation pipelines, optimizers, learning-rate schedules, and pretrained MMDetection (version 3.3.0) configurations were retained. The comparison therefore concerns complete detector configurations under shared resource and evaluation constraints; it is not a controlled architectural ablation in which network topology is the only changing factor.

All sonar-domain images used for model adaptation, validation, and held-out testing were acquired during real underwater measurement missions. No simulated, synthetically generated, style-transferred, or semi-synthetic sonar imagery was included in the sonar dataset. Although the detector parameters were initialized from models pretrained on the optical COCO dataset, all subsequent sonar-domain adaptation and evaluation were performed using real SSS measurements.

The measurements were acquired using the Gavia Defence AUV (Teledyne Gavia ehf., Kópavogur, Iceland) and the Light Autonomous Underwater Vehicle (LAUV; OceanScan–Marine Systems & Technology, Lda., Leça da Palmeira, Matosinhos, Portugal), both equipped with the same primary sonar model, the EdgeTech 2205 (EdgeTech, West Wareham, MA, USA). Three physical mine-like reference objects two Manta-type objects and one cylindrical object were imaged during multiple survey missions and repeated passes conducted under heterogeneous environmental and operational conditions. The measurements encompassed variations in water depth, seabed type and local bottom morphology, carrier platform, vehicle altitude above the seabed, sonar range, survey trajectory, target-to-track offset, target aspect, insonification side, acquisition date, and direction of approach. These factors were observed jointly rather than manipulated independently, and their variability was retained to represent realistic changes in sonar-contact appearance.

The development detection task comprised Manta-type reference objects, cylindrical reference objects, and non-target seafloor objects. Patches without an accepted bounding-box annotation were retained as negative examples and could contain seabed background, acoustic noise, or visually non-distinct structures. Two complete measurement missions were excluded in their entirety from model development and reserved as Test A and Test B. No raw sonar record, rendered image, extracted patch, annotation, or derivative image product originating from these missions was used during training, validation, augmentation design, hyperparameter selection, early stopping, checkpoint selection, or confidence-threshold determination. The held-out tests therefore assessed transfer to new acoustic observations, seabed contexts, survey trajectories, and mission-level acquisition conditions. Because the same physical reference-object designs were represented across the measurement campaigns, the evaluation should not be interpreted as a test of generalization to previously unseen mine designs.

The main contributions of this work are threefold: (i) a systematic comparison of five complete detector configurations representing two-stage, dense one-stage, multi-scale one-stage, anchor-free, and transformer-based detection paradigms under a shared dataset, training budget, effective batch size, checkpoint-selection rule, and evaluation protocol; (ii) an evaluation based exclusively on real sonar-domain measurements acquired during multiple missions by two AUV platforms equipped with the same primary sonar model, including mine-like reference targets, heterogeneous non-target contacts, and patches without accepted bounding-box annotations; and (iii) a separate evaluation on two complete survey missions withheld from all stages of model development, supplemented by analyses of class-wise detection performance, localization errors, missed detections, false-target alarms, confidence-dependent behavior, training convergence, computational cost, GPU-memory demand, and patch-level inference speed.

## 2. Materials and Methods

### 2.1. Study Design and Experimental Workflow

This study was designed as a systematic comparison of five complete deep object-detector configurations for automatic target recognition in real-world side-scan sonar imagery.

To clarify the scope of this work, the study does not propose a new neural network architecture. Instead, it presents an original empirical comparison of five established detector configurations using newly acquired real-world sonar data and independent complete-mission evaluation. The contribution is therefore experimental and methodological rather than the introduction of a new detection architecture. The evaluated configurations were based on Faster R-CNN, RetinaNet, SSD, YOLOX, and DETR and represented different object-detection paradigms.

Because each configuration retained its architecture-specific input policy, augmentation pipeline, optimizer, learning-rate schedule, and pretrained initialization, the experiment should not be interpreted as a controlled architectural ablation in which network topology was the only changing factor.

The operational unit of annotation, detector input, and patch-level evaluation was a fixed-size 200×200-pixel sonar-image patch. A patch could contain one or more annotated objects or contain no ground-truth bounding box. Patch counts and annotated-instance counts do not represent equal numbers of statistically independent physical objects. Multiple non-identical patches, repeated survey passes, and different acoustic-channel observations could correspond to the same physical reference contact and were therefore treated as related data products rather than independent target encounters.

**Figure 1:**
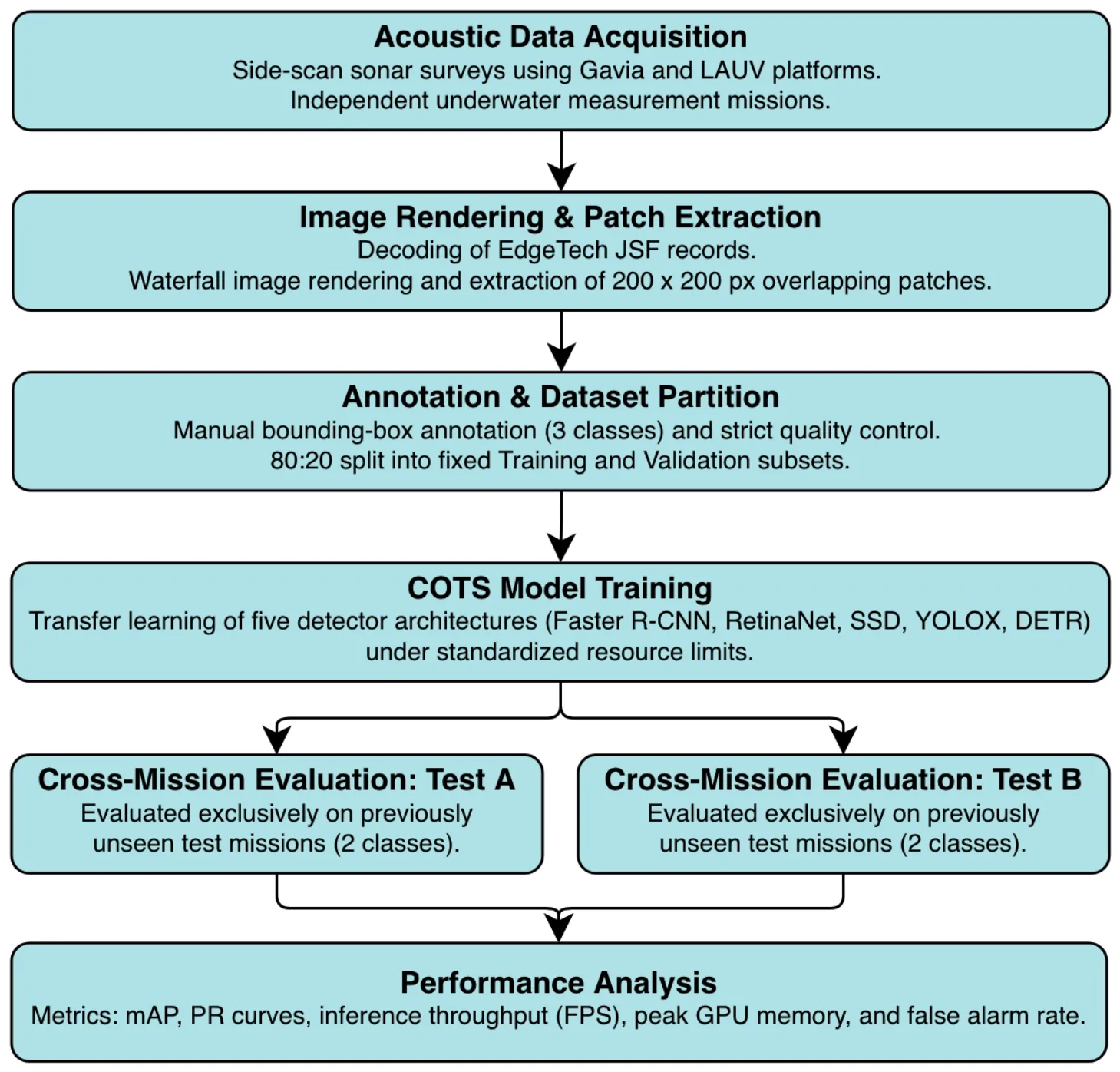
Flowchart of the proposed experimental workflow, from acoustic data acquisition to cross-mission evaluation and performance analysis.

The experimental workflow comprised: (i) acquisition of acoustic data during multiple AUV survey missions; (ii) decoding and rendering of native EdgeTech JSF records; (iii) extraction of fixed-size sonar-image patches; (iv) manual bounding-box annotation and quality control; (v) construction of fixed training and validation subsets using data originating exclusively from five development missions; (vi) transfer learning and optimization of the five detector configurations; and (vii) final cross-mission evaluation using two complete measurement missions excluded in their entirety from model development. The complete experimental workflow is illustrated in Figure 1.

The accepted development dataset was divided once, at patch level, into fixed training and validation subsets. The training subset was used for parameter optimization, whereas the validation subset was used for early stopping and checkpoint selection. The same development partition was used for every detector configuration. Because the partition was performed at patch level, related crops or channel-specific observations of the same physical contact were not explicitly grouped during the split. The possible dependence between such observations is considered when interpreting the validation results and is stated as a limitation of the study.

The two held-out missions were designated Test A and Test B and were evaluated separately. No raw sonar record, rendered image, extracted patch, annotation, or derivative image product originating from either test mission was used during training, validation, augmentation design, hyperparameter selection, early stopping, checkpoint selection, or confidence-threshold determination. The separation therefore prevented direct reuse of test-mission imagery during model development.

All sonar-domain images used in this study originated from real underwater measurements. No simulated, synthetically generated, style-transferred, or semi-synthetic sonar images were included. Detector parameters were initialized from checkpoints pretrained on the optical Microsoft COCO dataset; however, all subsequent sonar-domain adaptation, validation, and held-out testing were performed using the real SSS data described in the following sections.

Three physical mine-like reference objects were used during the measurement campaigns: two Manta-type objects assigned to one detection class and one cylindrical object assigned to the second reference-target class. The held-out evaluation therefore measured transfer to previously unseen sonar records, seabed contexts, acquisition geometries, survey trajectories, and mission-level conditions involving these reference objects. It did not constitute an evaluation of generalization to previously unseen physical target designs.

The development detection task comprised three annotated categories: Manta-type reference objects, cylindrical reference objects, and non-target seafloor objects. The non-target category contained discernible natural or anthropogenic objects that did not belong to either reference-target class. Patches without a ground-truth bounding box were retained as negative examples and could contain seabed background, acoustic noise, or unannotated structures.

Because heterogeneous non-target objects were not exhaustively annotated in Test A and Test B, quantitative cross-mission evaluation was restricted to the Manta-type and cylindrical reference-target classes. Nevertheless, all available patches from both test missions were processed by every detector, including patches without an annotated reference target. Predictions produced on these patches were retained for the evaluation of false reference-target alarms rather than being removed from the test dataset. A patch without a reference-target annotation should therefore not automatically be interpreted as verified homogeneous seabed, because it could also contain an unannotated benign object.

One training run was performed for each detector configuration. The study therefore provides a descriptive comparison of the selected runs under a common dataset, training budget, effective batch size, checkpoint-selection rule, and evaluation protocol. It does not quantify variability across random initializations or establish statistically resolved differences between detector families. The rankings reported in the following sections should consequently be interpreted as observations for the evaluated configurations and missions.

### 2.2. AUV Platforms and Sonar Acquisition System

Two autonomous underwater vehicles served as sonar carrier platforms: the Gavia Defence AUV and the Light Autonomous Underwater Vehicle (LAUV). Both vehicles carried an EdgeTech 2205 side-scan sonar. The objective was not to compare the hydrodynamic, navigational, or endurance characteristics of the platforms, but to acquire real SSS data under a broader range of operational conditions while retaining the same primary sonar model. The use of two platforms preserved variability associated with vehicle dynamics, mission planning, trajectory execution, and insonification geometry without introducing a fundamentally different sonar technology.

#### 2.2.1. Gavia Defence AUV

**Figure 2:**
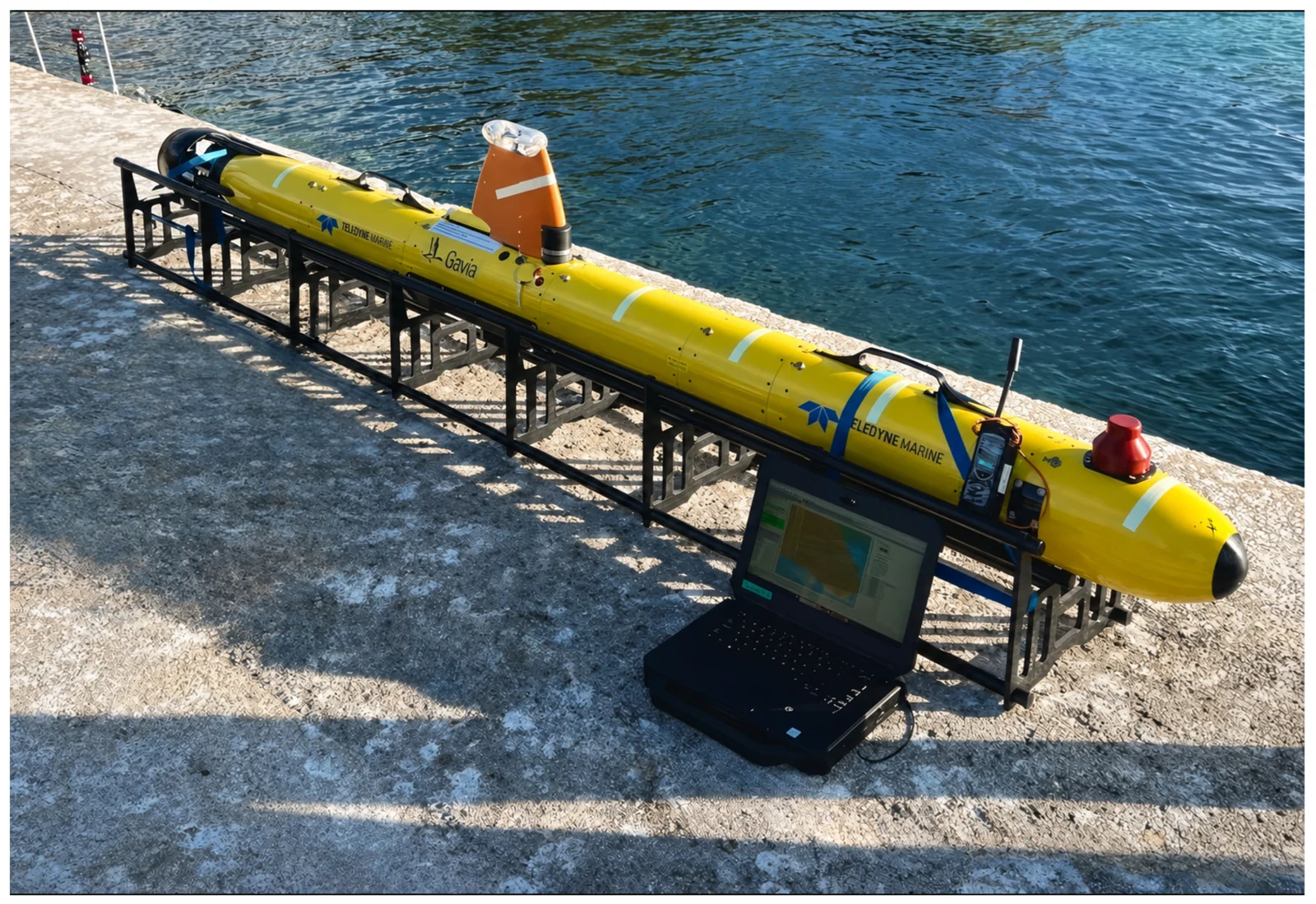
Gavia Defence AUV used as an autonomous carrier of the EdgeTech 2205 side-scan sonar during the measurement campaigns.

The Gavia Defence AUV shown in Figure 2 is a modular autonomous platform with a hull diameter of 200 mm. Depending on the installed modules, the Gavia platform is available with nominal depth ratings of 500 or 1000 m; the configuration used in this study was rated to 1000 m [27]. The experimental configuration had an approximate mass in air of 70 kg, although the exact mass depended on the installed payload and battery modules. Propulsion was provided by a stern-mounted screw propeller, while control surfaces provided horizontal and vertical maneuvering.

Surface positioning was based on GPS. During submerged operation, navigation and trajectory control were supported by a Doppler-velocity-log-aided inertial navigation system, supplemented by heading and pressure measurements. Mission planning and supervision were performed using Gavia Control Center (https://www.teledynemarine.com/brands/gavia/gavia-auv-software-and-data-handling, accessed on 12 October 2026), which was used to define waypoints, survey lines, payload settings, and safety criteria. Sonar and navigation records were stored onboard for post-mission processing.

A nominal commanded speed of 1.8 m/s was used along the SSS survey lines. This operating condition was selected to support stable trajectory execution and consistent along-track sampling during sonar acquisition.

#### 2.2.2. Light Autonomous Underwater Vehicle

**Figure 3:**
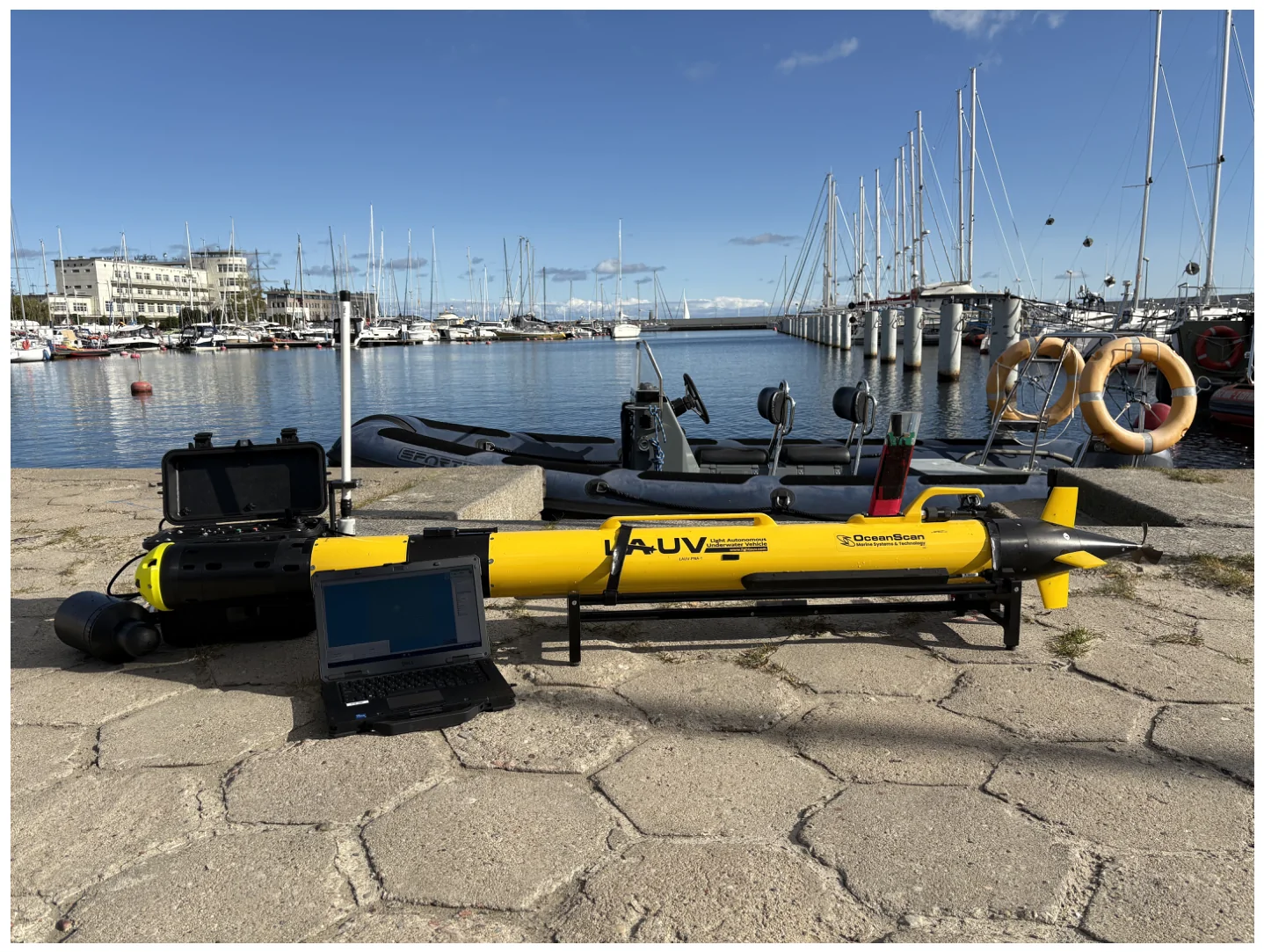
Light Autonomous Underwater Vehicle used as the second autonomous carrier of the EdgeTech 2205 side-scan sonar.

The LAUV shown in Figure 3 is a lightweight modular autonomous underwater vehicle developed by OceanScan–Marine Systems and Technology [28,29]. The configuration used in this study was approximately 2.3 m long, had a hull diameter of 0.15 m, an approximate mass in air of 32 kg, and a nominal maximum operating depth of 100 m.

Surface positioning was provided by GPS. During submerged operation, navigation and trajectory control were supported by an attitude and heading reference system, a pressure sensor, a Doppler velocity log, and acoustic-positioning support. The DVL provided bottom-referenced velocity and altitude measurements used by the navigation system during low-altitude survey operations.

Mission planning, payload configuration, execution monitoring, and post-mission review were performed using the Neptus command-and-control environment (software version not recorded; https://github.com/LSTS/neptus, accessed on 12 October 2026) [30]. Sonar and navigation records were stored onboard and retrieved after vehicle recovery. As in the Gavia surveys, a nominal commanded speed of 1.8 m/s was used along the SSS survey lines.

The principal characteristics of both study platforms are summarized in Table 1.

#### 2.2.3. EdgeTech 2205 Side-Scan Sonar

The acoustic data were acquired using EdgeTech 2205 side-scan sonar systems integrated with both AUV platforms. The EdgeTech 2205 is a modular and configurable acoustic system intended for installation on autonomous, remotely operated, and other hosted maritime platforms. It supports simultaneous multi-frequency operation and Full Spectrum CHIRP processing [31].

The installed configuration provided simultaneous low-frequency and high-frequency SSS channels with nominal center frequencies of approximately 600 and 1600 kHz, respectively. These values do not represent single monochromatic frequencies selected independently for individual targets. Within each channel, the sonar transmitted a frequency-modulated CHIRP pulse over a configured bandwidth. The instantaneous frequency varied throughout the pulse, and matched filtering of the received waveform compressed the pulse in time, improving range resolution while preserving transmitted acoustic energy. The values of approximately 600 and 1600 kHz therefore describe the nominal centers of the two broadband CHIRP channels.

The low-frequency channel provided a wider operational swath and greater effective range, whereas the high-frequency channel provided finer spatial detail over shorter ranges. Both channels could be recorded during the same survey pass and therefore represented correlated observations of the same insonified seabed scene rather than independent physical target encounters. The nominal characteristics of the installed transducer configuration are summarized in Table 2.

The sonar systems were integrated with the data-acquisition infrastructure of the respective AUV platforms. Timing information stored in the acquisition records provided a basis for associating sonar observations with the available vehicle-navigation data during post-mission processing. Because the two platforms used separate sonar units and integration architectures, the detailed synchronization and acoustic-interference-management arrangements were platform specific.

Raw acoustic measurements were stored in the native EdgeTech JSF format. A JSF file is a binary message-based structure in which acoustic data are organized according to subsystem, channel, and message type. Port and starboard SSS returns and the LF and HF channels can therefore be decoded separately. Depending on the integration, JSF records may also contain timestamps, navigation strings, attitude data, pressure measurements, and other auxiliary vehicle information [32].

The use of the same sonar model on both vehicles reduced sensor-model variability, but did not imply complete hardware identity because separate sonar units, vehicle integrations, and mission configurations could retain unit-specific or platform-specific differences.

### 2.3. Reference Objects, Environmental Variability, and Dataset Roles

Three full-scale mine-like reference objects were used during the measurement campaigns: two low-profile Manta-type objects and one elongated cylindrical object. The two Manta-type objects were assigned to the same detection class because they represented the same reference-target category, whereas the cylindrical object constituted the second mine-like target class.

The cylindrical reference target was a welded steel structure with a nominal outer diameter of 460 mm, an overall length of 1700 mm, a wall thickness of 12 mm, and a mass of approximately 350 kg. The Manta-type reference design had a nominal base diameter of 945 mm, a height of approximately 440 mm, and a mass of approximately 300 kg per object. These geometries represented two substantially different seabed-target profiles: a compact low-profile body and an elongated cylindrical body.

**Figure 4:**
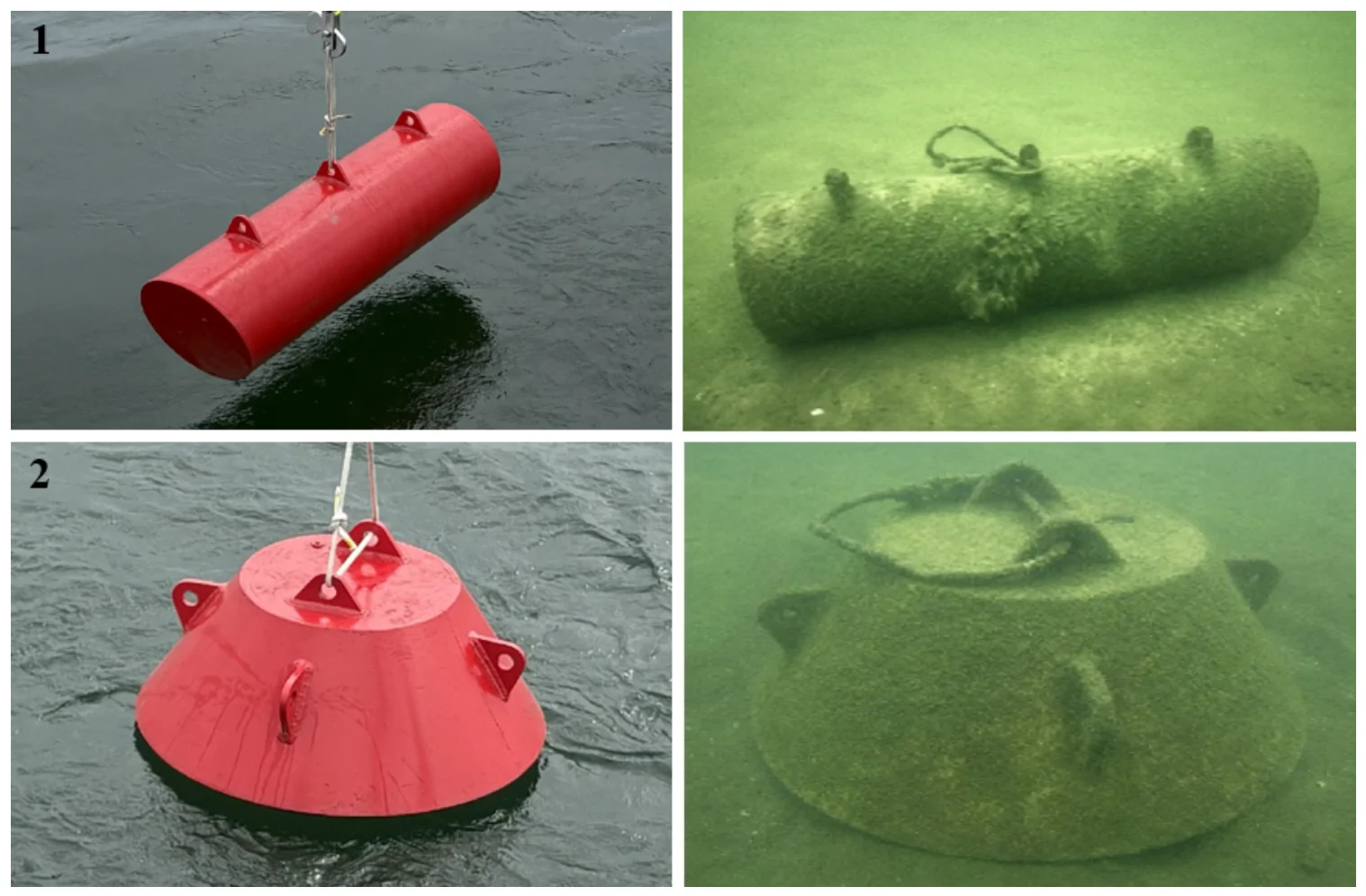
Reference objects used during the measurement campaigns. Panel (**1**) shows the cylindrical object, and panel (**2**) shows a Manta-type object. The (**left**) images show the objects during deployment, whereas the (**right**) images show them after placement on the seafloor.

The reference objects are shown in Figure 4, and their dimensions and class assignments are summarized in Table 3.

The sonar measurements were acquired during multiple AUV survey missions and repeated passes rather than during a single isolated reference campaign. Five complete survey missions supplied the development data, whereas two additional complete missions were withheld for independent cross-mission evaluation and designated Test A and Test B.

The reference objects were imaged under heterogeneous environmental and operational conditions. The measurements encompassed variations in water depth, seabed type and local bottom morphology, carrier platform, vehicle altitude above the seabed, sonar range, survey trajectory, target-to-track offset, target aspect, insonification side, acquisition date, and direction of approach.

These environmental and operational variables were not manipulated as independent experimental factors. Their natural variability was retained to increase the diversity of the recorded target signatures and to represent conditions encountered during realistic AUV-based SSS surveys. Accordingly, the present study does not attempt to quantify the isolated causal effects of water depth or seabed type on detector performance. Instead, these variables are treated collectively as sources of realistic cross-mission domain variability.

During the survey campaigns, predefined track lines and stable speed, depth, or altitude commands were used whenever operationally practicable. The known positions of the reference objects enabled repeated insonification from different directions, offsets, ranges, and aspect angles. During the relevant survey passes, the LF and HF CHIRP channels were recorded simultaneously. The paired channels represented different acoustic-resolution views of the same survey pass and were therefore treated as correlated data products rather than independent physical target encounters.

The missions were assigned to their respective dataset roles before detector development. Patches derived from the five development missions were used to construct the training and validation subsets. Test A and Test B were excluded in their entirety from model training, validation, early stopping, checkpoint selection, hyperparameter selection, and confidence-threshold determination, as specified in Section 2.1. The two test missions were evaluated separately rather than pooled, thereby allowing the stability of cross-mission generalization to be assessed under two distinct sets of mission-level conditions.

All available patches from both test missions, including patches without an annotated reference target, were processed by every detector. Because heterogeneous non-target objects were not exhaustively annotated, the non-target class was excluded from the quantitative test metrics. Manta-type and cylindrical predictions on patches without an annotated reference target were retained for the separate false-target analysis.

This experimental design evaluates generalization to previously unseen sonar records, seabed contexts, acquisition geometries, and mission-level conditions. Where the same physical reference exemplars occurred in both the development and test missions, independence concerned the recorded acoustic observations and mission conditions rather than the identity of the physical objects. Accordingly, the independent tests assess generalization to new acoustic observations and mission conditions rather than to novel physical target designs.

### 2.4. JSF Decoding, Image Rendering, and Patch Extraction

The native JSF records were decoded separately for the relevant subsystems, CHIRP channels, and port and starboard sides. Acoustic samples within each ping represented the across-track slant-range direction, whereas consecutive pings were arranged in acquisition order along the vehicle trajectory. This procedure generated two-dimensional waterfall images from the original acoustic records.

The development image dataset included five predefined visualization profiles associated with different survey records and processing configurations. Each profile combined a color mapping with software-level time-varied-gain and normalization settings. The values listed in Table 4 are processing-software parameters and should not be interpreted as calibrated target strength, absolute receive gain, or physical measurements of the acoustic response.

The visualization profiles altered the rendered image appearance but did not constitute additional acoustic measurements. All five object detectors received exactly the same prepared image files, and no model-specific rendering procedure was applied. No target was digitally inserted into an image, and no simulated, style-transferred, or semi-synthetic sonar scene was used. The networks therefore operated on rendered three-channel sonar imagery rather than on calibrated acoustic amplitude or absolute target-strength measurements. For each held-out test mission, the rendered patch set was fixed before detector evaluation. Within a given test mission, exactly the same rendered patches were supplied to all five detector configurations, and no detector- specific rendering adjustment was applied.

The visualization profiles applied to Test A and Test B were drawn from the same set of processing-software configurations listed in Table 4. Because the rendering profile determines the color mapping, gain, and normalization of the resulting image, any difference between the profile distribution of the development dataset and that of a test mission constitutes an additional, image-level component of the cross-mission domain shift. This factor was not manipulated experimentally and is considered when interpreting the between-mission differences reported in Section 3.

The rendered waterfall images were divided into fixed-size 200×200-pixel patches using a 100-pixel stride in both image directions, corresponding to 50% overlap between adjacent patches. Contact-containing patches retained the visible sonar contact together with a portion of the surrounding seabed. The overlapping extraction could therefore produce multiple non-identical patches containing the same physical contact at different image positions and with different local seabed contexts. Pixel-identical duplicate files were excluded during quality control.

Each patch was retained and evaluated as a separate image. No cross-patch spatial deduplication, georeferenced merging, or contact aggregation was applied. Consequently, related overlapping patches could contain repeated appearances of the same physical contact, and the reported patch-level detection counts should not be interpreted as counts of unique physical contacts.

### 2.5. Dataset Composition and Development Partition

Before quality control, the candidate development dataset comprised 12,783 fixed-size image patches. The patches were organized into three source groups associated with Manta-type contacts, cylindrical contacts, and non-target contacts. These source-group counts should not be interpreted as object-instance counts because an individual patch could contain multiple annotated objects, while some candidate patches ultimately contained no accepted bounding-box annotation.

Bounding boxes and class labels were prepared manually using the LabelImg annotation tool (software version not recorded; https://github.com/tzutalin/labelImg, accessed on 12 October 2026) [33] and stored in Pascal VOC XML format [34]. The annotations were subsequently converted to COCO format for detector training and evaluation. A bounding box was drawn around each visually distinguishable Manta-type or cylindrical reference target. Visually separable non-target contacts were annotated individually, whereas a dense group of non-target structures that could not be separated reliably was represented by one enclosing bounding box. Patches without an accepted object annotation were represented by empty annotation files and retained as negative samples.

Bounding-box annotations were prepared by two annotators. Each annotator’s work was reviewed by the other annotator, and labels or bounding boxes were corrected when the reviewers identified an inconsistency. This mutual cross-review was applied before the accepted development dataset and the two independent test datasets were finalized.

Quality control was performed before construction of the training–validation partition. A candidate patch was classified as ambiguous when the available rendered sonar appearance did not permit the presence of a distinct contact or its class membership to be determined with sufficient visual confidence. Such patches were excluded rather than assigned to the non-target class. This decision reduced the risk of introducing knowingly uncertain class labels, but it also restricted the resulting dataset to contacts above the adopted visual-interpretability threshold.

A total of 717 candidate patches, corresponding to 5.61% of the initial candidate dataset, were excluded. The exclusions comprised 648 Manta-associated patches, 47 cylindrical-associated patches, and 22 non-target-associated patches. The corresponding source-group exclusion rates were 14.52%, 1.91%, and 0.38%, respectively. The final development dataset therefore contained 12,066 accepted image–annotation pairs.

The class-specific quality-control flow is summarized in Table 5.

The substantially higher exclusion rate in the Manta-associated source group (14.52%) than in the cylindrical-associated group (1.91%) indicates that the adopted visual-confidence criterion affected the two reference-target source groups unequally. A plausible contributing factor is the difference in target geometry and the resulting sonar appearance. The Manta-type objects have a compact, low-profile geometry, whereas the cylindrical reference object is elongated and protrudes more prominently from the seabed. Consequently, under less favorable insonification geometries, target-to-track offsets, seabed conditions, or rendering settings, some Manta-type contacts could produce a less distinctive highlight–shadow pattern and become more difficult to identify confidently in an individual 200×200-pixel patch.

In addition, the Manta-associated source group contained observations of two physical exemplars, compared with one cylindrical exemplar, and therefore incorporated a broader set of target appearances across repeated passes and mission conditions. These factors provide plausible explanations for the observed difference, but they cannot be separated quantitatively in the present dataset. Structured rejection-reason categories were not assigned to individual excluded patches; therefore, the higher Manta exclusion rate cannot be attributed conclusively to target geometry, aspect, burial, seabed type, signal quality, or image-rendering settings. The excluded patches were removed before construction of the training–validation partition and were not used during training or evaluation. Consequently, the reported results characterize contacts that satisfied the adopted visual-interpretability criterion and do not quantify detector performance on the ambiguous cases.

Of the 12,066 accepted patches, 1764 contained no bounding-box annotation and were retained as negative samples. The remaining 10,302 patches contained at least one annotated object. Patches without an annotation therefore represented 14.62% of the final development dataset. Depending on their source imagery, these patches could contain seabed background, acoustic noise, or visually non-distinct structures; they should not be interpreted as independently verified examples of uniform seabed.

The accepted development dataset contained 18,253 annotated object instances: 3778 Manta-type instances, 2400 cylindrical instances, and 12,075 non-target instances. These values represent annotated sonar appearances in image patches rather than independent physical objects or independent target encounters. In particular, the Manta-type and cylindrical instance counts were derived from repeated survey passes, channel-specific observations, and shifted patch extractions involving two physical Manta-type reference objects and one physical cylindrical reference object.

The resulting class-wise instance counts are summarized in Table 6.

A custom stratified splitting script divided the accepted development patches once, at patch level, into training and validation subsets in an 80:20 proportion. Stratification accounted for the three annotated categories and the patches without annotations so that their representation was preserved as closely as possible in both subsets. A pseudorandom seed of 42 was used solely to make this data partition reproducible. It did not control model initialization or other stochastic training operations, for which separately generated seeds were used in the individual detector runs.

The training subset contained 9652 patches and 14,687 annotated instances, whereas the validation subset contained 2414 patches and 3566 annotated instances. The resulting partition was frozen and used without modification for all five detector configurations.

The exact COCO annotation files defining the training and validation membership of this frozen partition are included in the Supplementary reproducibility package.

Because the development partition was constructed at patch level, related observations of the same physical contact could occur in both the training and validation subsets. These related observations included repeated survey passes, channel-specific views, and spatially shifted non-identical patches derived from the same physical reference objects. The validation subset should therefore be interpreted as a within-development model-selection set rather than as an independent test of physical-target or mission-level generalization. Independent generalization was assessed exclusively using Test A and Test B, whose complete source missions were excluded from all stages of model development.

The final training and validation partition is summarized in Table 7.

Pixel-identical duplicate image files were excluded before construction of the development partition. However, the available metadata were not used to enforce grouping at the level of a physical reference object, survey pass, acoustic contact, or paired LF/HF observation. Because the split was performed at patch level, non-identical shifted crops or repeated sonar observations associated with the same physical reference object could occur in both the training and validation subsets. Such patches could contain the contact at different positions or against different local seabed contexts, but they cannot be regarded as statistically independent physical target exemplars.

For this reason, validation results were interpreted as within-dataset development performance and were used for early stopping and checkpoint selection. They were not treated as the principal evidence of cross-mission performance. Two consequences follow and should be stated explicitly. First, because overlapping patches extracted with a 100-pixel stride may place near-duplicate views of the same physical contact in both subsets, the reported validation ${\mathrm{mAP}}_{50:95}$ is expected to be optimistic relative to performance on genuinely independent data. Second, this optimism is not confined to reporting: the same validation metric governed early stopping and checkpoint selection, so the selected checkpoints were chosen under a partially dependent criterion. This consideration is relevant when interpreting the divergence between the validation ranking and the cross-mission rankings reported in Section 3. Evaluation beyond the development dataset was performed separately on Test A and Test B, each originating from a complete measurement mission excluded from training, validation, checkpoint selection, hyperparameter selection, and confidence-threshold determination.

Because the trained models were generated from this fixed patch-level development partition, a physical-target-, acoustic-contact-, or mission-grouped validation result could not be obtained retrospectively without redefining the development split and retraining all five detector configurations. Instead, the dependence of the independent-mission results on validation-based checkpoint selection was assessed through a retrospective checkpoint-sensitivity analysis. This analysis does not remove the statistical dependence within the development partition, but provides a direct check of whether the reported Test A and Test B results are strongly dependent on the particular checkpoint selected by the within-development validation metric.

No formal inter-annotator agreement statistic or sensitivity analysis of the excluded ambiguous patches was included in the present study. These omissions limit the extent to which annotation uncertainty and the influence of the adopted visual-confidence criterion can be quantified. They are therefore considered explicitly when interpreting the reported performance.

### 2.6. Object-Detection Architectures and Evaluated Configurations

Five object-detection architectures were selected to represent substantially different detection paradigms rather than incremental variants of a single model family. Faster R-CNN represented two-stage detection based on a region proposal network [22]. RetinaNet represented dense one-stage detection and used focal loss to reduce the contribution of easily classified background examples [23]. SSD represented computationally efficient one-stage detection based on predictions from multiple feature-map scales [24]. YOLOX represented anchor-free one-stage detection with a decoupled prediction head and dynamic label assignment [25]. DETR represented transformer-based direct set prediction using encoder–decoder attention and bipartite matching [26].

The concrete implementations evaluated in this study were Faster R-CNN with a ResNet-50 backbone and feature pyramid network, RetinaNet with ResNet-50 and a feature pyramid network, SSD300 with a VGG-based backbone, the small YOLOX variant (YOLOX-S), and DETR with a ResNet-50 backbone. These variants corresponded to the respective COCO-pretrained MMDetection configurations used to initialize the detectors.

The selected detector variants and their detection paradigms are summarized in Table 8.

All models were implemented in MMDetection 3.3.0 using Python 3.10.11, PyTorch 2.1.0 with CUDA 11.8, MMEngine 0.10.7, MMCV 2.1.0, NumPy 1.26.4, and OpenCV 4.9.0.80 [35,36]. The same software environment and workstation were used for all five configurations. A software and hardware environment record extracted from the original MMEngine training logs is included in the Supplementary reproducibility package.

Detector parameters were initialized from checkpoints pretrained on the Microsoft COCO dataset [37]. Output layers whose dimensions depended on the number of object categories were adapted from the 80 COCO categories to the three sonar-domain categories. Parameters that were incompatible with the modified output dimensions were initialized for the new detection task. COCO pretraining was used only for parameter initialization; all subsequent sonar-domain optimization, validation, checkpoint selection, and held-out testing used the real SSS data described in the preceding sections.

The selected MMDetection configurations retained their architecture-specific backbones, input-resizing policies, augmentation pipelines, optimizers, initial learning rates, and learning-rate schedules. These components were not forced to identical numerical settings because they form part of the established training pipeline of each selected detector. In particular, the five configurations did not use a common input resolution or a common data-augmentation pipeline.

Experimental comparability was instead established through the use of the same training and validation patches, the same three sonar-domain training classes, a common maximum budget of 50 epochs, the same effective batch size of eight, the same validation-based early-stopping criterion, and the same checkpoint-selection metric. All configurations were subsequently evaluated using the same held-out test missions and the same evaluation implementation.

A single final training run was performed for each detector configuration. Model-training seeds were not fixed to one common value; each run used an automatically generated seed recorded in its training log. Consequently, the experiment does not quantify variation across random initializations, and small numerical differences between configurations should not be interpreted as statistically resolved architectural effects.

The results therefore describe five complete detector configurations evaluated under common data, resource-budget, checkpoint-selection, and evaluation constraints. They do not constitute an architectural ablation in which network topology was the only changing experimental variable. Configuration-specific input dimensions, data augmentations, and batch implementation are documented in the following subsection. Optimizers and learning-rate schedules followed the corresponding MMDetection configuration families and were intentionally not harmonized.

### 2.7. Training Protocol

The maximum training budget was fixed at 50 epochs for every detector configuration. Early stopping monitored the three-class validation bounding-box ${\mathrm{mAP}}_{50:95}$, reported by MMDetection as coco/bbox_mAP. Training was terminated after 10 consecutive validation epochs without an improvement of at least 0.001. The checkpoint corresponding to the highest observed validation ${\mathrm{mAP}}_{50:95}$ was selected separately for each configuration and subsequently used without modification for evaluation on Test A and Test B.

Checkpoint selection was governed exclusively by validation performance. The held-out test missions were not used to select an epoch, compare intermediate checkpoints, modify the training schedule, or optimize a confidence threshold. At most three periodic checkpoint files were retained at any time to limit disk usage. This storage setting should not be interpreted as preserving exactly the three highest-scoring checkpoints.

To quantify the sensitivity of the independent-mission results to this validation-based checkpoint choice without retraining the detectors, a retrospective checkpoint-sensitivity analysis was performed. For each configuration, the checkpoint selected by the highest validation ${\mathrm{mAP}}_{50:95}$ was compared with one prespecified alternative: the terminal checkpoint reached by the original training run. The validation-selected and terminal epochs were 15 and 25 for Faster R-CNN, 47 and 50 for YOLOX, 10 and 20 for RetinaNet, 40 and 50 for SSD, and 32 and 42 for DETR, respectively. The terminal checkpoint was defined solely by the original training termination and was not selected on the basis of Test A or Test B performance. Both checkpoints were evaluated using the same frozen detector configuration and the same two-class COCO evaluation protocol. The primary test results reported in this study remain those obtained with the validation-selected checkpoints; the terminal-checkpoint results are reported only as a post-hoc sensitivity analysis and were not used for model selection or threshold tuning.

The effective batch size was fixed at eight images for all five configurations. SSD, RetinaNet, and YOLOX used a physical batch size of eight without gradient accumulation. Faster R-CNN and DETR used a physical batch size of four with a gradient-accumulation factor of two to remain within the available GPU-memory capacity. Consequently, the configurations performed approximately the same number of optimizer updates per epoch despite differences in their logged numbers of physical iterations.

All configurations received the same original 200×200-pixel sonar patches, but their model-input resizing and augmentation policies were retained from their respective MMDetection configuration families. The resulting settings are summarized in Table 9.

Background-only patches were retained during training using the following MMDetection dataset-filter configuration:

filter_cfg = dict(filter_empty_gt = False, min_size = 1)

This setting prevented patches without ground-truth bounding boxes from being removed by the dataset filter. All five configurations were therefore exposed during optimization to negative examples containing seabed background, acoustic noise, or other structures without an accepted object annotation.

The architecture-specific input policies resulted in different numbers of input pixels processed per patch. Consequently, measured differences in inference throughput, GPU-memory allocation, and training time reflect the complete detector pipelines, including their resizing and augmentation policies, rather than network topology alone. In particular, the reported FPS values should not be interpreted as a controlled comparison at an identical model-input resolution.

The selected configurations also retained their architecture-specific optimizers, initial learning rates, learning-rate schedules, loss functions, assignment strategies, and post-processing procedures. These settings were not forced to identical numerical values because the five detectors rely on different established optimization and prediction mechanisms. Faster R-CNN and RetinaNet used proposal- or anchor-based processing, SSD used multi-scale anchor-based prediction, YOLOX used dynamic assignment within an anchor-free pipeline, and DETR used bipartite matching and direct set prediction.

The same frozen training and validation partitions, the same three sonar-domain training categories, the same maximum epoch budget, the same effective batch size, the same early-stopping rule, and the same checkpoint-selection metric were applied to all configurations. Accordingly, the experiment standardized the principal data, resource, and model-selection constraints while retaining the native configuration-specific training pipelines.

One final training run was performed for each configuration. The training–validation partition was generated once using the fixed pseudorandom seed of 42 and was subsequently reused unchanged for all five detector configurations. Runtime training seeds were generated separately for the individual MMEngine runs and were preserved in the corresponding original training logs. The recorded seed information is included in the Supplementary reproducibility package. Because repeated multi-seed training was not performed, the reported between-model differences are treated as descriptive outcomes of the evaluated runs rather than estimates of run-to-run variability.

All training runs were performed on a workstation running Windows 10 and equipped with a single NVIDIA GeForce GTX 1070 GPU (NVIDIA Corporation, Santa Clara, CA, USA) with 8 GB of video memory. The reported wall-clock training times, peak CUDA memory allocations reported by MMEngine, and patch-level inference rates are specific to this hardware and software environment. They do not directly establish performance, latency, energy consumption, or real-time capability on an embedded AUV computing platform.

Peak memory values were extracted uniformly from the memory field reported in the standardized MMEngine training logs for all five configurations. In MMEngine, this field corresponds to PyTorch’s max_memory_allocated statistic and is reported in MB. It therefore represents the maximum GPU memory occupied by tensors as tracked by the PyTorch allocator, rather than an independent measurement of total physical VRAM residency.

For DETR, the maximum recorded value was 9509 MB. This value exceeds the nominal 8 GB of dedicated VRAM of the NVIDIA GeForce GTX 1070 used in the experiment. The workstation operated under Windows 10 and the Windows Display Driver Model (WDDM), which provides GPU virtual-memory and residency management. However, the MMEngine training log did not record the physical residency of individual allocations or the division between local GPU memory and system-memory backing at the time of the reported peak. Consequently, the value of 9509 MB should not be interpreted as 9509 MB simultaneously resident in the physical 8 GB VRAM of the GTX 1070. It is retained as the MMEngine/PyTorch allocator-reported tensor-memory statistic. The same MMEngine log field and the same extraction criterion were used for all five detector configurations.

The common 50-epoch limit represents a standardized, resource-constrained comparative budget. Validation-based early stopping reduced unnecessary optimization after performance ceased to improve under the adopted criterion; however, the resulting checkpoints should not be interpreted as evidence that every detector reached its architecture-specific asymptotic optimum.

One aspect of this shared budget warrants explicit comment. DETR optimizes a set-prediction objective through bipartite matching, and the original work adopts training schedules substantially longer than the limit applied here: 300 epochs for the reported ablation experiments, with the learning rate reduced after 200 epochs, and 500 epochs for the comparison against Faster R-CNN, with the learning rate reduced after 400 epochs [26]. The 50-epoch limit used in the present study therefore corresponds to between one sixth and one tenth of the schedules under which this architecture was originally characterized. The DETR results reported in this study should accordingly be read as performance under a shared, resource-constrained budget, and not as an estimate of the accuracy attainable by transformer-based set prediction on this dataset under a schedule matched to its convergence behavior.

The present experimental design does not include a controlled ablation in which all detectors are retrained using an identical model-input resolution and data-augmentation policy, nor does it include a DETR-specific extended-training-budget ablation. Consequently, all between-model comparisons reported in this study are interpreted at the level of the complete evaluated detector configurations. Differences in detection accuracy, computational efficiency, and cross-mission behavior cannot be attributed causally to the detection paradigm or network topology alone.

### 2.8. Evaluation Protocol

Detection performance was evaluated using the COCO bounding-box protocol [37]. For a predicted bounding box $B_{p}$ and a ground-truth bounding box $B_{g}$, intersection over union was calculated as

(1) $$\mathrm{IoU}\left(B_{p},B_{g}\right)=\frac{\left|B_{p}\cap B_{g}\right|}{\left|B_{p}\cup B_{g}\right|}.$$

The primary performance measure was COCO bounding-box mean average precision, averaged over IoU thresholds from 0.50 to 0.95 in increments of 0.05 and denoted as ${\mathrm{mAP}}_{50:95}$. Secondary COCO measures included ${\mathrm{mAP}}_{50}$, ${\mathrm{mAP}}_{75}$, class-wise average precision, and average recall with a maximum of 100 detections per image, denoted as ${\mathrm{AR}}_{100}$. All five detector configurations were evaluated using the same COCO-format annotations and the same evaluation implementation.

Average precision was additionally reported for the three COCO object-size categories, denoted ${\mathrm{AP}}_{S}$, ${\mathrm{AP}}_{M}$, and ${\mathrm{AP}}_{L}$. These categories are defined by absolute bounding-box area: below $32^{2}$ pixels, between $32^{2}$ and $96^{2}$ pixels, and above $96^{2}$ pixels, respectively [37]. Because the evaluated patches measure 200×200 pixels, these thresholds correspond to approximately 2.6% and 23.0% of the patch area, so an instance assigned to the COCO large category occupies close to one quarter of the patch. The size-based values reported in Section 3 should therefore be interpreted with reference to the patch geometry adopted in this study rather than to the absolute object scales typical of natural-image benchmarks.

COCO average precision was calculated from predictions ordered by their confidence scores and was therefore not associated with one fixed operating confidence threshold. Predictions were retained from a low confidence threshold of 0.01 for the confidence-ranked COCO evaluation, thereby reducing premature removal of low-confidence predictions before construction of the precision–recall sequence. The separate confidence threshold of 0.30 described below was used only for operating-point diagnostics and false-target summaries; it did not affect the reported COCO AP values.

Validation performance was evaluated over all three development categories: Manta-type reference objects, cylindrical reference objects, and non-target seafloor objects. The three-class validation ${\mathrm{mAP}}_{50:95}$ was used for early stopping and checkpoint selection. The selected checkpoint for each detector configuration was then frozen and applied separately to Test A and Test B.

Both test missions had been excluded in their entirety from training, validation, early stopping, checkpoint selection, hyperparameter selection, augmentation design, and confidence-threshold determination. No checkpoint or threshold was selected separately for either test mission after inspection of its results. The subsequent terminal-checkpoint sensitivity evaluation was retrospective and did not alter the validation-selected checkpoints used for the primary Test A and Test B results.

Because heterogeneous non-target seafloor objects were not exhaustively annotated in the independent missions, these patches may contain seabed background, acoustic clutter, or unannotated natural or anthropogenic non-target objects. The reported values therefore quantify false reference-target alarms under the adopted annotation protocol rather than a false-positive rate measured on exhaustively verified object-free seabed.

A COCO image record was created for every available test patch, irrespective of whether the patch had an associated target annotation. Patches without a Manta-type or cylindrical annotation were represented without a reference-target bounding box. All available patches were processed during inference. Target-class predictions on patches without an annotated reference target were therefore retained in the evaluation rather than being removed before metric calculation.

Three related but distinct evaluation scopes were used:

1. Confidence-ranked COCO metrics were calculated for the two reference-target classes using all test patches and the complete set of retained target-class predictions.
2. Threshold-dependent precision, recall, F1-score, and confusion matrices were calculated on patches containing an annotated reference target. These diagnostics characterized classification and localization behavior in the vicinity of known targets at a common operating threshold.
3. False-target behavior outside the annotated target patches was summarized separately over patches without a Manta-type or cylindrical ground-truth annotation.

This separation prevents the very large number of patches without an annotated target from obscuring the interpretation of target-localization and target-classification diagnostics, while still reporting their false-target burden separately. Consequently, the precision, recall, and F1-score values at confidence 0.30 should be interpreted together with the false-detections-per-patch values and not as standalone mission-level alarm rates.

For the threshold-dependent target diagnostics, a common confidence threshold of 0.30 was applied to every detector configuration and both test missions. Predictions and ground-truth boxes were matched one-to-one using an IoU threshold of 0.50. Matching was performed greedily in descending order of prediction confidence. A matched prediction was counted as a true positive (TP) when its assigned class agreed with the ground-truth class. A prediction matched spatially to a ground-truth object but assigned to the wrong reference-target class was represented as a class confusion. Unmatched target-class predictions within this diagnostic scope were counted as false positives (FP), whereas unmatched ground-truth objects were counted as false negatives (FN).

For each target class, precision, recall, and F1-score were calculated as

(2) $$P=\frac{TP}{TP+FP},$$

(3) $$R=\frac{TP}{TP+FN},$$

and

(4) $$F1=\frac{2PR}{P+R},$$

respectively. These measures describe performance at the fixed confidence threshold of 0.30 and IoU threshold of 0.50. They are distinct from COCO AP, which integrates confidence-ranked behavior over multiple recall levels and, for the primary metric, multiple IoU thresholds.

The threshold of 0.30 was adopted as a common diagnostic operating point and was not optimized separately for any detector or test mission. It therefore enabled consistent calculation of the reported diagnostic tables but did not guarantee an equivalent recall, false-alarm rate, or calibration level across configurations. Results at this threshold should not be interpreted as a calibrated operational comparison.

False-target behavior was characterized as both an absolute count and the mean number of Manta-type and cylindrical predictions per patch without an annotated reference target. For a test mission containing $N_{\text{no-target}}$ such patches, the normalized measure was calculated as

(5) $${\mathrm{FP}}_{\mathrm{patch}}=\frac{N_{\mathrm{false}\;\mathrm{target}\;\mathrm{detections}}}{N_{\text{no-target}}}.$$

Only Manta-type and cylindrical predictions above the confidence threshold of 0.30 were included in this false-target summary. Predictions assigned to the incompletely annotated non-target class were excluded.

A patch without a Manta-type or cylindrical annotation should not necessarily be interpreted as physically verified uniform seabed. Such a patch could contain seabed background, acoustic noise, clutter, or an unannotated natural or anthropogenic non-target object. The reported quantity therefore measures false reference-target alarms under the adopted annotation protocol rather than detections on exhaustively verified object-free imagery.

False detections were counted at patch level after the native per-image post-processing of each detector. No cross-patch spatial deduplication, contact aggregation, or georeferenced merging was applied. Consequently, predictions generated for the same physical feature in multiple related or overlapping patches could contribute more than once to the reported counts. The values should therefore not be interpreted as numbers of unique physical false contacts, false contacts per unit survey area, or operator-review events.

Test A and Test B were evaluated and reported separately. Their predictions, annotations, and false-target counts were not pooled into a single test dataset. This retained the observed differences between the two acquisition missions and avoided allowing the substantially larger Test B dataset to dominate a pooled metric.

The primary validation metric covered three classes, whereas the aggregate Test A and Test B metrics covered only the two reference-target classes. The original three-class validation and two-class test values therefore represent different category sets and are not directly comparable. Accordingly, validation performance was additionally recalculated at the same selected checkpoints using only the Manta-type and cylindrical reference-target classes. These class-matched validation results are reported separately in Section 3.2 and provide a more appropriate descriptive baseline for comparison with the independent mission tests. Nevertheless, differences in mission conditions and sample sizes remain, so the resulting values should not be interpreted as repeated measurements from the same data distribution.

Test A contained 21 annotated reference targets, whereas Test B contained 251. Because of the small number of targets in Test A, individual detections, localization errors, or missed targets can have a comparatively large influence on its aggregate result. To quantify test-set sampling uncertainty without retraining the detector configurations, a non-parametric bootstrap resampling analysis was performed on the existing network predictions for Test A and Test B. For each test, N=1000 bootstrap replicates were generated by sampling image patches with replacement (seed = 42). The bounding-box mAP was evaluated exclusively for the two reference-target classes on each replicate, yielding a 95% percentile confidence interval [2.5th, 97.5th percentiles] associated with the point estimates. The resampling was performed at the image patch level rather than grouped by physical contact because reliable identifiers linking overlapping non-identical patches to the underlying physical target could not be extracted retrospectively from the test split files.

Patch-level inference time was measured for every detector using the same evaluation script and workstation. Mean latency per patch and its reciprocal in frames per second were reported. These measurements characterize the implemented patch-level pipelines on the desktop GTX 1070 workstation. They do not include evaluation of the complete onboard processing chain, including sonar-record decoding, waterfall rendering, patch generation, cross-patch contact aggregation, georeferencing, navigation-data processing, communication, or operator interaction.

## 3. Results

### 3.1. Training Convergence and Computational Cost

The five detector configurations exhibited different convergence profiles under the common maximum budget of 50 epochs. Faster R-CNN, RetinaNet, and DETR terminated through early stopping after 25, 20, and 42 epochs, respectively. SSD and YOLOX completed the full 50-epoch budget. The selected checkpoints occurred at epochs 15, 10, and 32 for Faster R-CNN, RetinaNet, and DETR, and at epochs 40 and 47 for SSD and YOLOX, respectively.

**Figure 5:**
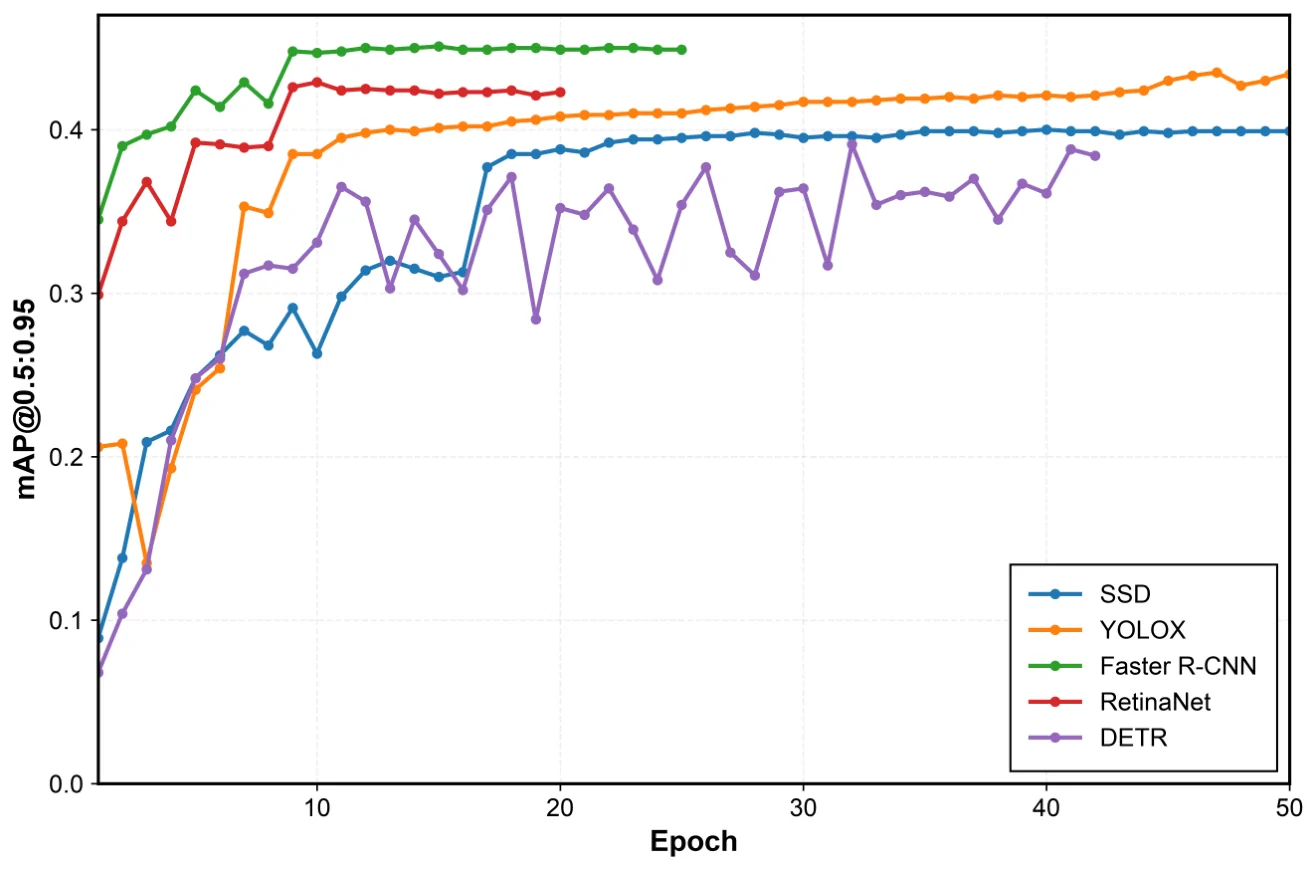
Evolution of three-class validation ${\mathrm{mAP}}_{50:95}$ during training. Curves end when early stopping was triggered or the 50-epoch budget was reached.

Figure 5 presents the three-class validation ${\mathrm{mAP}}_{50:95}$ recorded after each completed epoch. Faster R-CNN reached its highest value relatively early and then remained close to its maximum until early stopping. RetinaNet followed a similar pattern over a shorter run. SSD improved more gradually and stabilized near 0.400, whereas YOLOX continued to show small fluctuations through the final epochs. DETR displayed the largest epoch-to-epoch variation among the five configurations.

Table 10 summarizes the selected epoch, total training length, wall-clock time, and peak allocator-reported tensor-memory statistic extracted from the MMEngine training logs. SSD required the shortest training time (approximately 7.18 h) and had the lowest reported peak memory value (5473 MB). DETR required the longest training time (approximately 33.07 h) and had the highest reported peak memory value (9509 MB). These computational values are specific to the single-GPU workstation described in Section 2.7.

**Figure 6:**
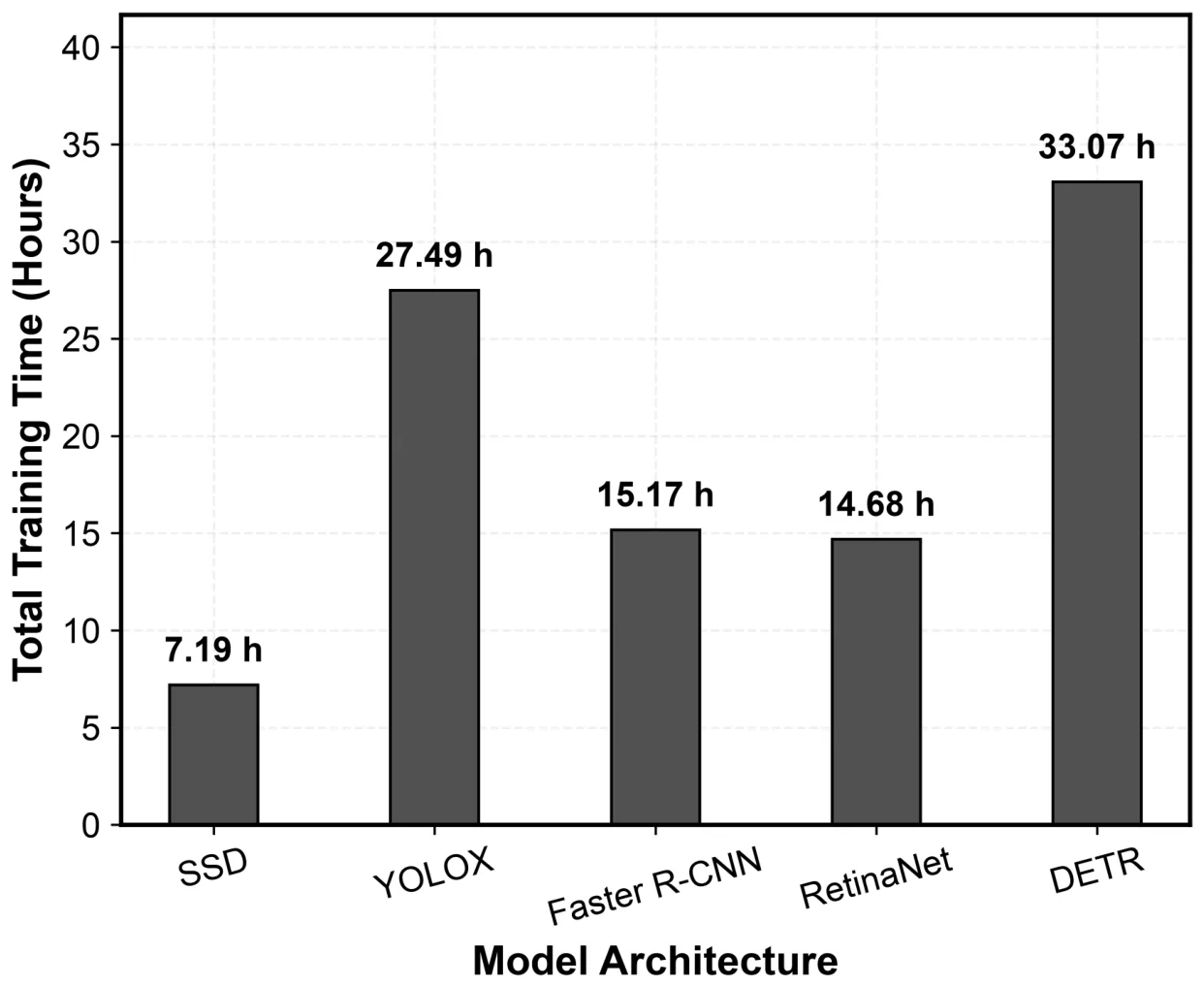
Wall-clock training time for the final detector runs on the common GTX 1070 workstation.

The wall-clock comparison is shown graphically in Figure 6. The differences reflect complete architecture-specific configurations, including their native input and augmentation pipelines, and should not be attributed to network topology alone.

### 3.2. Detection Performance on the Validation Subset

Table 11 reports the metrics obtained at the checkpoint selected separately for each architecture. Faster R-CNN achieved the highest three-class validation ${\mathrm{mAP}}_{50:95}$ (0.451), followed by YOLOX (0.435), RetinaNet (0.429), SSD (0.400), and DETR (0.391). The difference between the highest and lowest values was 0.060. All five models achieved substantially higher ${\mathrm{mAP}}_{50}$ than ${\mathrm{mAP}}_{75}$, indicating that bounding-box localization became a major source of error as the IoU requirement increased.

To provide a class-matched baseline for the independent mission tests, Table 12 reports the validation metrics recalculated at the same selected checkpoints using only the Manta-type and cylindrical reference-target classes. Faster R-CNN achieved the highest two-class validation ${\mathrm{mAP}}_{50:95}$ of 0.568, followed by YOLOX (0.547), RetinaNet (0.537), SSD (0.508), and DETR (0.497).

The object-size values in Table 11 were calculated at the selected checkpoint rather than by taking an independent maximum for each size category. Medium-object AP was the largest size-based value for SSD, Faster R-CNN, YOLOX, and DETR. RetinaNet instead achieved its largest value for the COCO large-object category. YOLOX showed the largest within-model difference across object-size categories, with ${\mathrm{AP}}_{L}=0.209$ compared with ${\mathrm{AP}}_{M}=0.444$.

### 3.3. Independent Cross-Mission Test Results

The two independent missions differed substantially in size and in the number of annotated reference targets. Test A contained 35,672 patches and 21 annotated targets, whereas Test B contained 68,741 patches and 251 annotated targets. All patches were processed during inference. Table 13 summarizes the target-class ground truth used for quantitative evaluation.

The aggregate and class-wise COCO results are reported in Table 14. These metrics cover only the two exhaustively annotated reference-target classes. On Test A, SSD achieved the highest ${\mathrm{mAP}}_{50:95}$ (0.614), followed by DETR (0.330), YOLOX (0.289), RetinaNet (0.098), and Faster R-CNN (0.064). SSD also obtained the highest class-wise AP for both the Manta-type (0.470) and cylindrical (0.758) classes.

The ranking changed on Test B. YOLOX achieved the highest ${\mathrm{mAP}}_{50:95}$ (0.084), followed by Faster R-CNN (0.071), SSD (0.065), DETR (0.060), and RetinaNet (0.033). The absolute values were much lower and more tightly grouped than in Test A. YOLOX obtained the highest Manta-type AP (0.111), whereas Faster R-CNN obtained the highest cylindrical AP (0.082). No architecture ranked first on both independent missions.

#### Checkpoint-Selection Sensitivity

A retrospective sensitivity analysis was performed to assess how strongly the independent-mission results depended on the checkpoint selected using the within-development validation metric. Table 15 compares the primary results obtained with the validation-selected checkpoint with those obtained using the terminal checkpoint reached by the same training run. The terminal checkpoints were not selected using either independent test mission.

The sensitivity differed substantially between the two independent missions. On Test A, the change in ${\mathrm{mAP}}_{50:95}$ ranged from +0.006 for Faster R-CNN to +0.091 for RetinaNet, while DETR decreased by 0.074. SSD remained the highest-performing configuration, although YOLOX and DETR exchanged their relative positions. In contrast, the Test B changes were small, ranging in absolute magnitude from 0.001 to 0.010, and the complete configuration ranking remained unchanged. Thus, the leading configuration on each mission was stable to this alternative checkpoint choice, but the Test A point estimates and part of the within-mission ordering showed appreciable checkpoint sensitivity.

**Figure 7:**
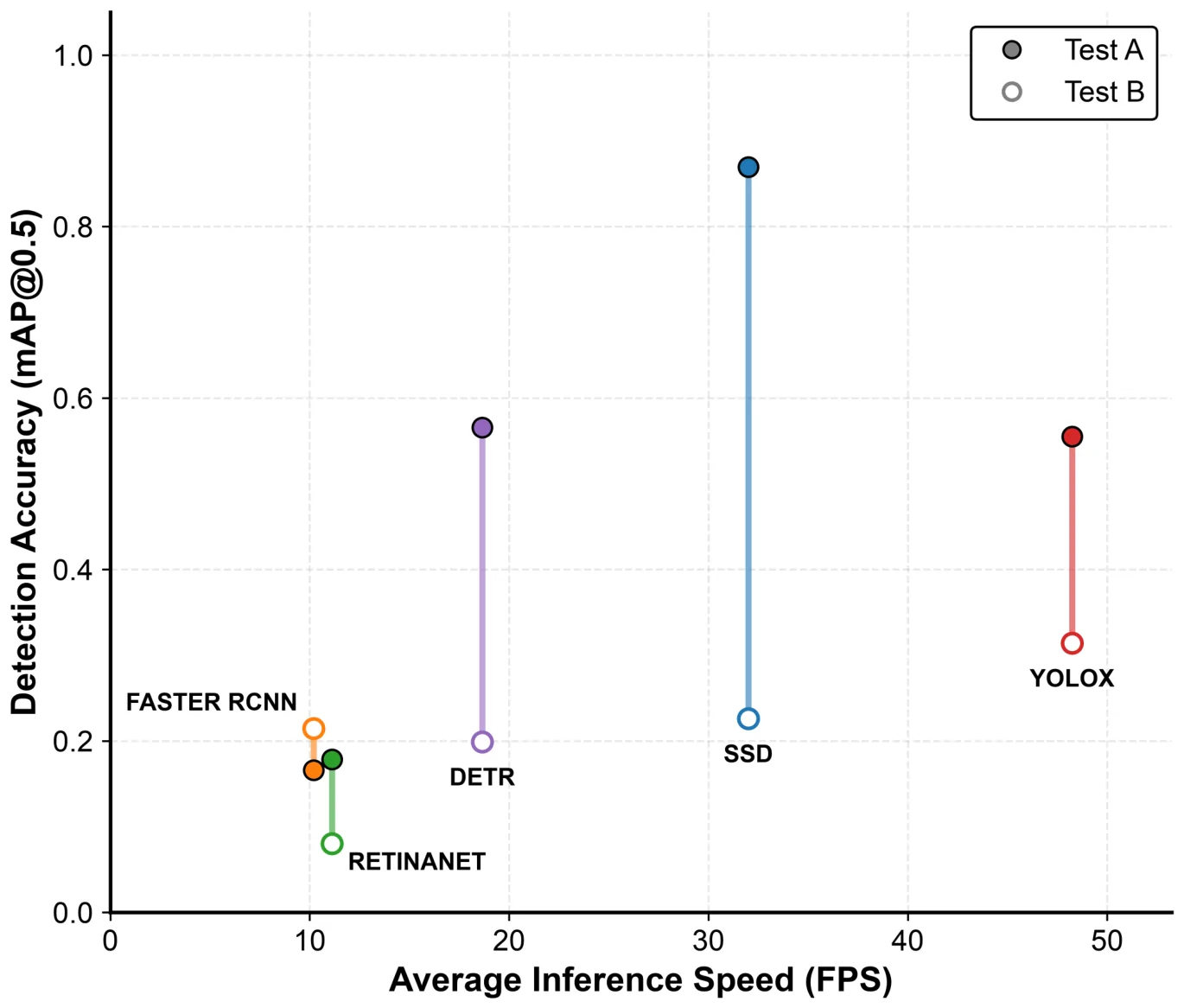
Trade-off between two-class ${\mathrm{mAP}}_{50}$ and average inference throughput on the two test missions. Each line joins the two mission results for one detector architecture.

Figure 7 complements the primary ${\mathrm{mAP}}_{50:95}$ values in Table 14 by showing the operating trade-off between ${\mathrm{mAP}}_{50}$ and inference throughput. The plot must therefore not be read as a comparison between validation and test performance.

### 3.4. Threshold-Dependent Target Detection Analysis

Table 16 reports precision, recall, and F1-score at the common diagnostic confidence threshold of 0.30 and an IoU matching threshold of 0.50. These values describe one operating point and are distinct from the confidence-ranked COCO AP results.

On Test A, all five architectures detected every cylindrical target at this operating point, but cylindrical precision did not exceed 0.030 because of the large number of unmatched cylindrical predictions. Manta-type recall was 1.000 for SSD, YOLOX, and DETR, but the associated precision ranged from 0.012 for DETR to 0.205 for SSD. Faster R-CNN obtained the highest Manta-type precision (0.455) but detected only one-third of the annotated Manta-type targets.

On Test B, YOLOX achieved the highest Manta-type F1-score (0.450), followed by SSD (0.413). Faster R-CNN achieved the highest cylindrical F1-score, but the value remained only 0.091. Cylindrical precision did not exceed 0.052 for any architecture, showing that cylindrical false alarms were a persistent error mode at the selected operating threshold.

**Figure 8:**
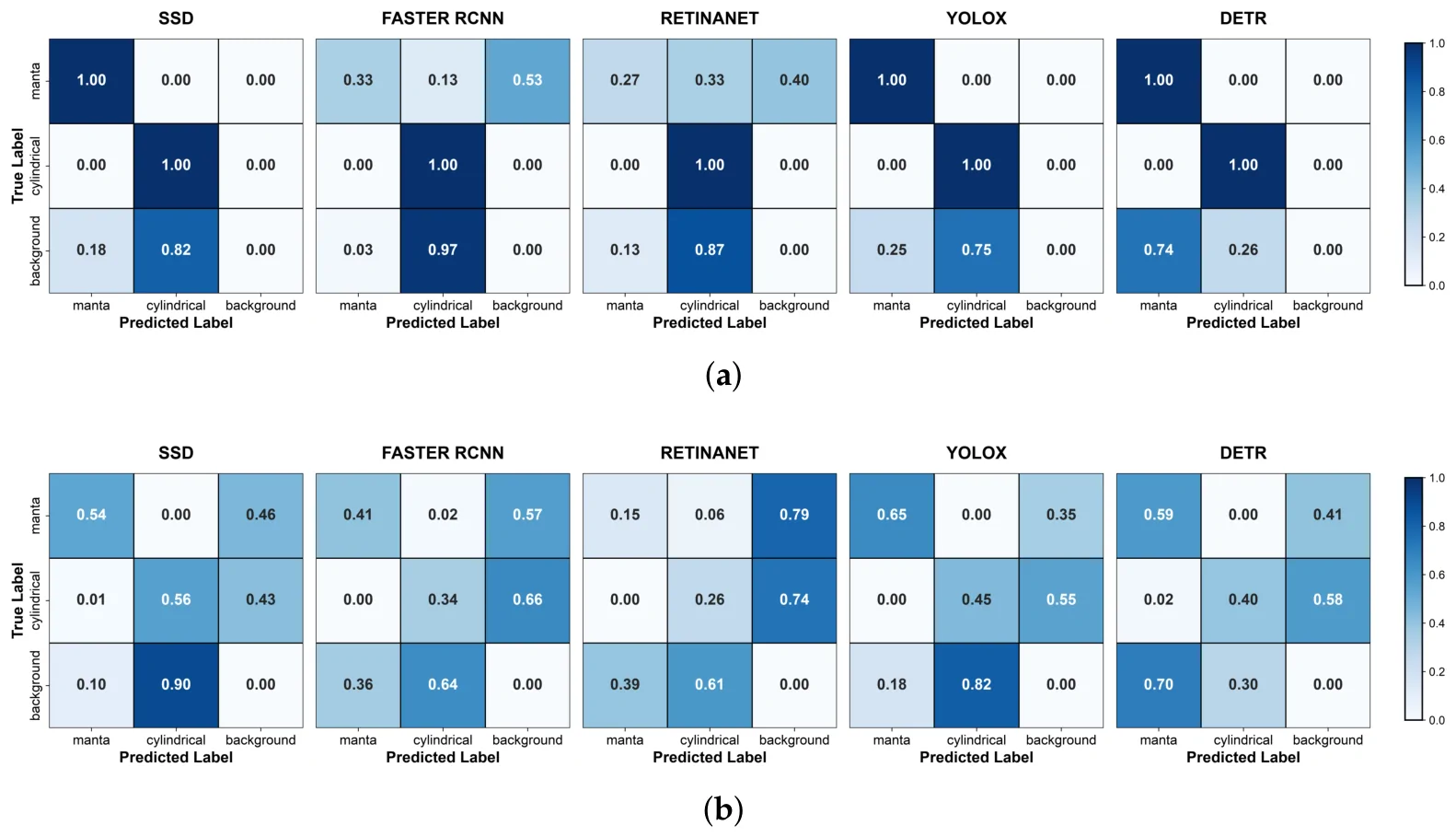
Row-normalized target-class confusion matrices at confidence 0.30 and IoU 0.50: (**a**) Test A and (**b**) Test B. The class *other* is excluded because it was not exhaustively annotated in the independent missions.

The row-normalized confusion matrices are shown in Figure 8. Their background rows describe the class distribution of unmatched predictions and do not convey the absolute false-alarm burden; absolute false-target counts and per-patch values are reported in Section 3.5.

### 3.5. False-Alarm Behavior and Inference Throughput

False-target behavior was quantified over patches without an annotated Manta-type or cylindrical target. YOLOX produced the lowest mean number of false target detections per such patch in both missions: 0.148 on Test A and 0.253 on Test B. SSD ranked second, with corresponding values of 0.257 and 0.460. DETR produced the largest values in both tests, reaching 0.947 on Test A and 1.467 on Test B. The value increased from Test A to Test B for every detector configuration.

Because heterogeneous non-target seafloor objects were not exhaustively annotated, these values quantify false reference-target alarms under the adopted annotation protocol. They should not be interpreted as detections on physically verified object-free seabed.

**Figure 9:**
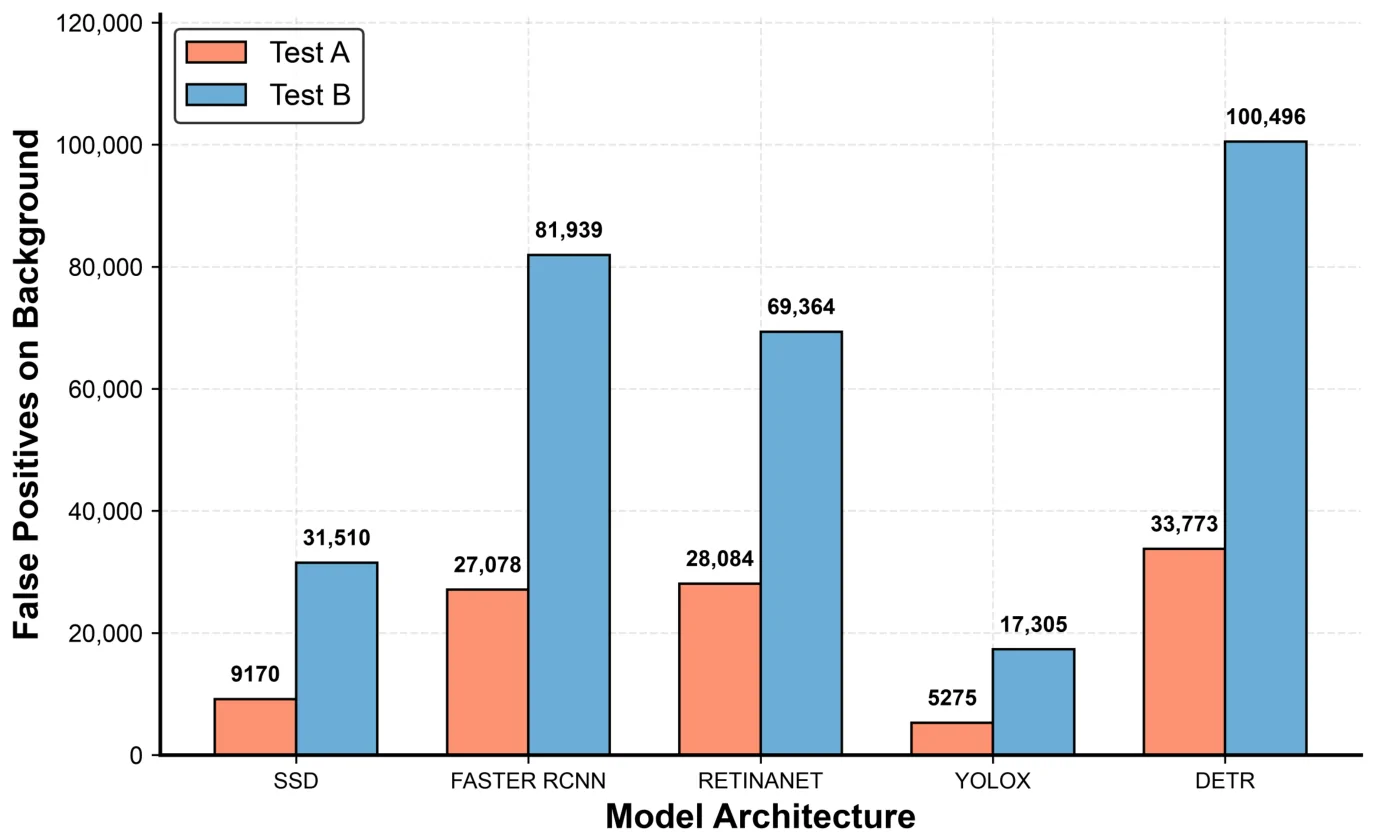
Absolute numbers of false Manta-type and cylindrical detections on patches without an annotated reference target.

Figure 9 shows the corresponding absolute numbers of false detections. Because Test A and Test B contained different numbers of patches, cross-mission comparison should use the normalized false-detections-per-patch values reported in Table 17 rather than bar height alone. The term “Background” in the vertical-axis label denotes patches without an annotated reference target and does not imply independently verified object-free seabed.

Inference throughput was consistent across the two missions for each architecture, as also visible in Figure 7. YOLOX was the fastest detector, reaching 47.13 FPS on Test A and 49.35 FPS on Test B. SSD processed approximately 32 FPS, DETR approximately 19 FPS, and RetinaNet approximately 11 FPS. Faster R-CNN was the slowest configuration, processing approximately 10 FPS. These values were measured on the same GTX 1070 workstation and do not represent performance on an embedded AUV computer.

### 3.6. Qualitative Detection Examples

**Figure 10:**
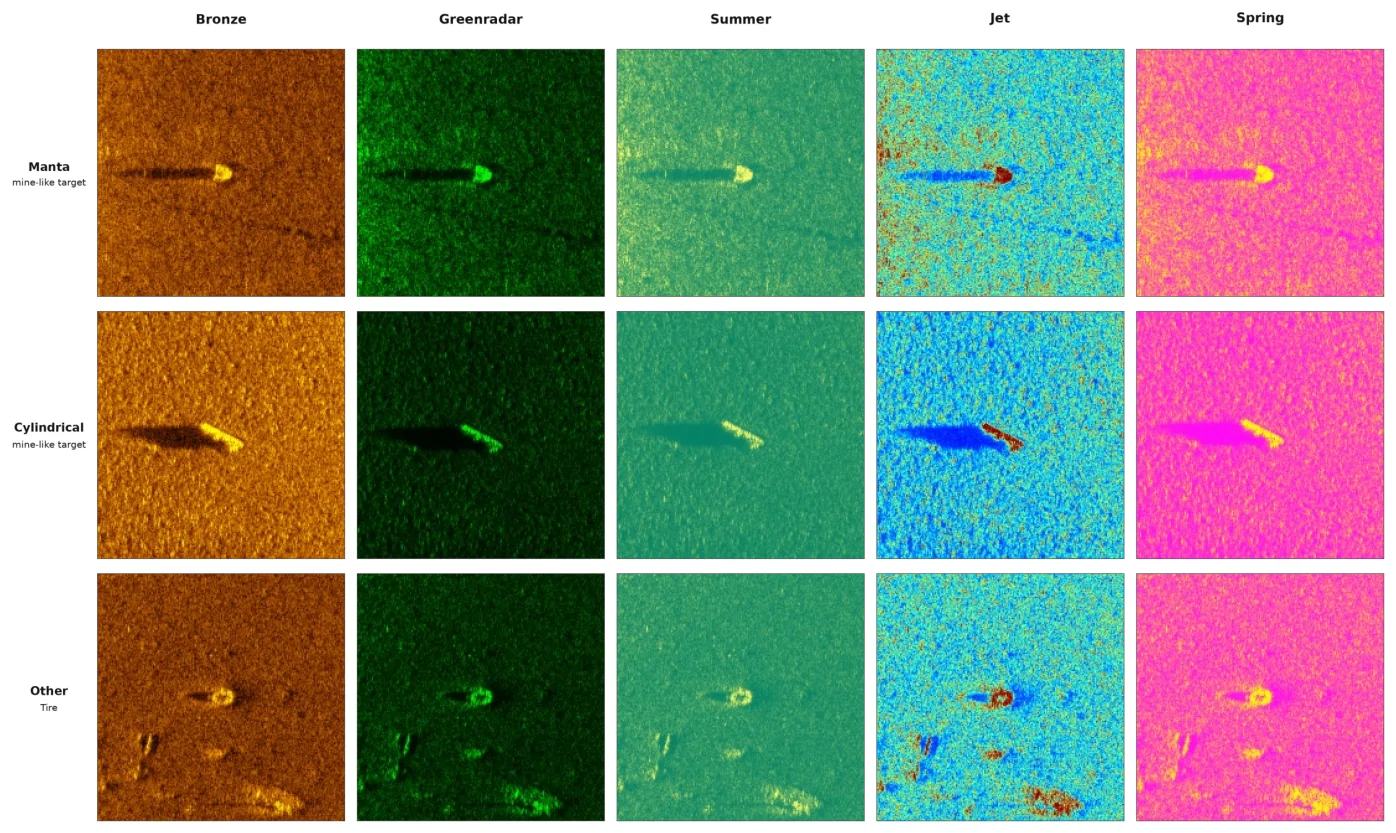
Representative development-dataset patches for the three annotated classes across the five visualization profiles. The columns show different representative patches and should not be interpreted as repeated renderings of one identical acoustic observation.

Figure 10 illustrates the appearance of representative Manta-type, cylindrical, and non-target contacts across the five visualization profiles present in the development dataset. The examples show substantial variation in color mapping, seabed texture, highlight appearance, and acoustic-shadow contrast.

**Figure 11:**
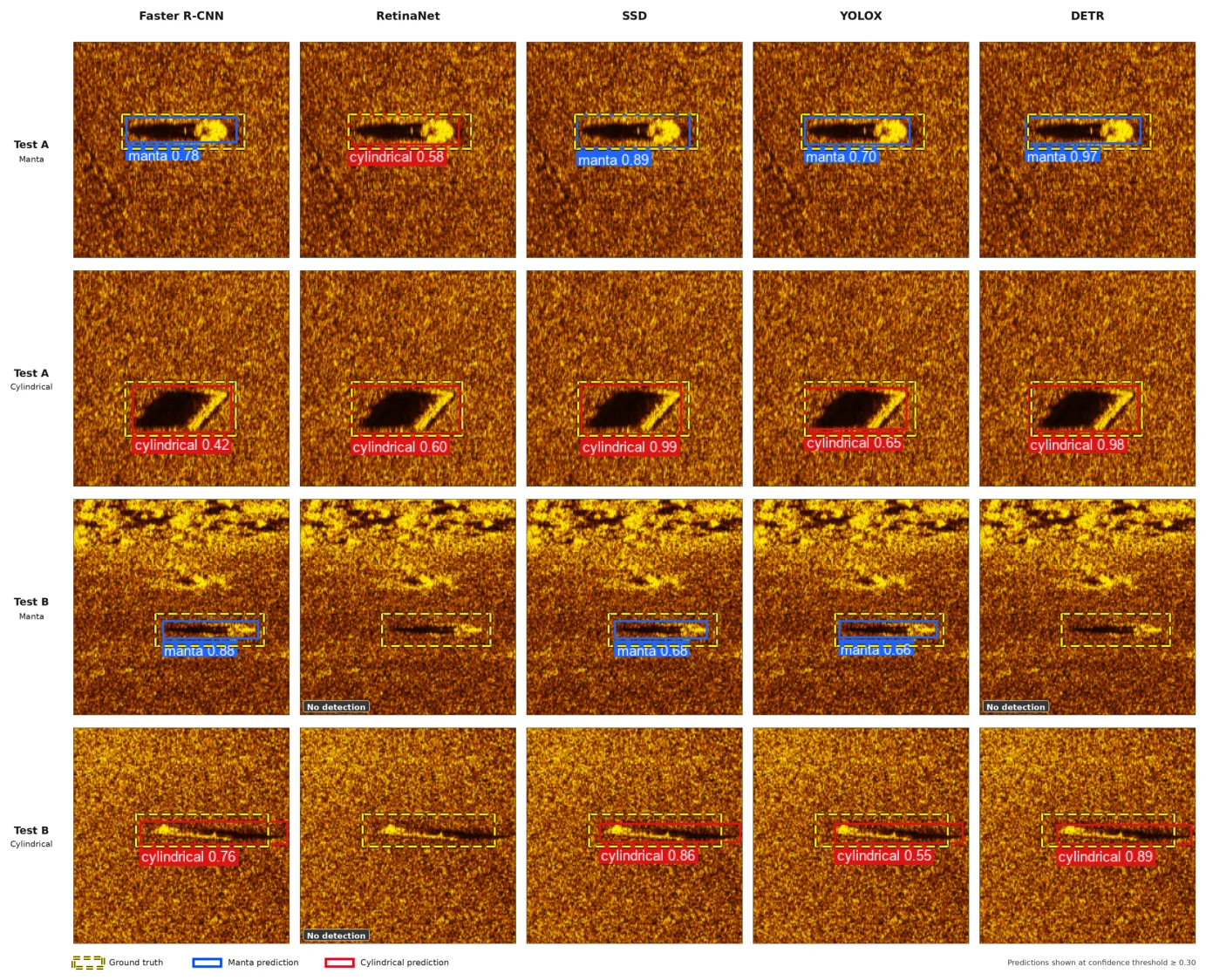
Qualitative detections from all five architectures on selected Test A and Test B patches. Yellow dashed boxes denote ground truth, blue boxes denote Manta-type predictions, and red boxes denote cylindrical predictions. Predictions are displayed at confidence 0.30.

Figure 11 compares the outputs of all five detectors on selected Manta-type and cylindrical examples from both independent missions. The Test A examples were detected by most architectures, although RetinaNet assigned the cylindrical label to the illustrated Manta-type contact. The selected Test B examples show greater cross-model disagreement: RetinaNet and DETR missed the illustrated Manta-type contact, while RetinaNet also missed the illustrated cylindrical contact. These examples are qualitative illustrations and were not used to select checkpoints or thresholds.

Overall, the validation ranking did not predict a single detector configuration that dominated both independent missions. Faster R-CNN achieved the highest three-class validation result, SSD achieved the highest two-class Test A result, and YOLOX achieved the highest two-class Test B result together with the fewest false target detections per patch without an annotated reference target and the highest inference throughput in both missions. These mission-dependent differences form the basis for the cross-mission analysis in Section 4.

## 4. Discussion

### 4.1. Cross-Mission Performance and Model Ranking

The principal finding of this study is that validation performance did not identify a detector that dominated both independent test missions. Faster R-CNN achieved the highest validation ${\mathrm{mAP}}_{50:95}$ under both three-class (0.451) and two-class (0.568) evaluations, whereas SSD achieved the highest two-class ${\mathrm{mAP}}_{50:95}$ on Test A (0.614) and YOLOX achieved the highest value on Test B (0.084). This change in ranking demonstrates that model selection based only on the within-dataset validation subset would not have predicted the best-performing configuration for either test mission.

The retrospective checkpoint-sensitivity analysis further showed that the cross-mission point estimates were not invariant to checkpoint choice. On Test A, replacing the validation-selected checkpoint with the terminal checkpoint changed ${\mathrm{mAP}}_{50:95}$ by +0.029 for SSD, +0.006 for Faster R-CNN, +0.091 for RetinaNet, +0.010 for YOLOX, and −0.074 for DETR. SSD remained the highest-performing configuration, although YOLOX and DETR exchanged their relative positions. On Test B, the corresponding changes were −0.001, −0.002, +0.001, +0.004, and −0.010, respectively, and the complete ranking remained unchanged. These findings indicate that checkpoint selection can materially affect individual cross-mission point estimates, particularly on the smaller Test A, while the identity of the highest-performing configuration on each independent mission remained stable. The validation ranking should therefore be interpreted as a within-development model-selection result rather than as evidence of independent generalization.

The contrast between the two independent tests was substantial even though the same detector checkpoints and evaluation implementation were used. SSD reached ${\mathrm{mAP}}_{50:95}=0.614$ on Test A, including the highest AP for both reference-target classes, but its corresponding aggregate on Test B was 0.065. YOLOX ranked first on Test B with ${\mathrm{mAP}}_{50:95}=0.084$, while its Test A result was 0.289. All five configurations produced much lower and more closely grouped values on Test B than on Test A. These observations are consistent with a strong mission-dependent change in the difficulty and statistical characteristics of the sonar imagery.

This mission dependence is expected in SSS imagery, where the appearance of a contact is jointly affected by the seabed, target aspect, target-to-track offset, grazing geometry, acoustic shadow, platform trajectory, sensor range, and image-rendering conditions [5,6]. The present experiment did not manipulate these factors independently and therefore cannot identify a single cause of the difference between Test A and Test B. The results should instead be interpreted as the combined effect of realistic cross-mission variability. This interpretation is also consistent with reviews that identify robustness across environments, sensors, and datasets as a persistent limitation of sonar ATR [2,3,10,21].

The different sizes of the two test missions critically affect the certainty with which their results can be interpreted. Test A contained only 21 annotated reference targets, compared with 251 in Test B. Consequently, the bootstrap confidence intervals for Test A are markedly wider, indicating high metric volatility where the presence or absence of a single target heavily influences the result. The strong performance of SSD on Test A should therefore be interpreted strictly as a descriptive outcome for this specific limited mission, rather than a statistically resolved ranking that generalises robustly across conditions where confidence intervals overlap.

### 4.2. Configuration-Level Accuracy–Efficiency Trade-Offs

The results do not support selection of one detector configuration solely from its validation AP. Instead, configuration selection depends on the intended operating conditions and the relative importance of localization quality, inference speed, memory demand, and false-alarm burden. SSD produced the strongest result on Test A while requiring the shortest training time and the lowest MMEngine-reported peak memory value among the evaluated configurations. It also sustained approximately 32 FPS on both test missions. Under conditions resembling Test A, SSD therefore offered the most favorable combination of detection quality and moderate computational demand.

YOLOX provided the most consistent operational trade-off across the two test missions. It ranked first on Test B, processed 47.13 FPS on Test A and 49.35 FPS on Test B, and produced the lowest mean number of false target detections per patch without an annotated reference target in both tests. These properties make it the strongest candidate in the present comparison for high-throughput contact screening. The finding is directionally consistent with the emphasis on one-stage and lightweight detectors in recent real-time SSS research [16,17,18,20]. However, the measured throughput concerns patch-level inference on a desktop GTX 1070 and does not by itself establish real-time operation on an embedded AUV computer.

Faster R-CNN illustrates the risk of relying on within-dataset validation alone. Although it obtained the highest validation ${\mathrm{mAP}}_{50:95}$, it ranked last on Test A and second on Test B, with an inference rate of approximately 10 FPS. DETR achieved the second-highest aggregate on Test A but required the longest training time, the greatest MMEngine-reported peak memory value, and produced the largest mean numbers of false target detections per patch without an annotated reference target in both missions. RetinaNet did not achieve the highest aggregate result in any of the three evaluation settings. These findings apply to the specific detector configurations, training protocol, and mission data evaluated in this study.

### 4.3. False Alarms and Class-Dependent Failure Modes

False alarms were a major limitation at the common diagnostic confidence threshold of 0.30. On Test A, every detector recalled all six cylindrical targets at IoU 0.50, yet cylindrical precision did not exceed 0.030. On Test B, cylindrical precision did not exceed 0.052 and the highest cylindrical F1-score was only 0.091. High recall at this operating point therefore did not imply operationally useful selectivity. These results identify cylindrical detections as a major source of false-reference-target alarms at the adopted operating point.

Retaining patches without accepted bounding-box annotations during training was methodologically important because it exposed every detector to seabed-background and acoustic-noise examples. Nevertheless, the test results show that this measure alone was insufficient to control the false-alarm burden across missions. YOLOX yielded the lowest values, with 0.148 and 0.253 false target detections per patch without an annotated reference target on Test A and Test B, respectively. Even these values would create a substantial number of contacts for human review over a complete survey. DETR produced the largest corresponding values, reaching 0.947 and 1.467.

The false-alarm analysis must also be interpreted in light of the annotation policy. Non-target seafloor objects were not exhaustively annotated in the two test missions, and the non-target class was consequently excluded from their aggregate metrics. A patch without a Manta-type or cylindrical annotation was therefore not guaranteed to contain only homogeneous seabed. The reported quantity measures false reference-target alarms under the available annotation protocol, rather than detections on independently verified object-free imagery. Exhaustive annotation of hard non-target contacts would be required to distinguish pure background activations from confusion with real but benign objects.

The operating-point diagnostics also show why COCO AP and threshold-dependent precision should be reported together. COCO AP summarizes confidence-ranked predictions over multiple recall levels, whereas the confusion matrices and false-alarm summaries describe performance at one practical threshold. The common threshold of 0.30 enabled a consistent comparison, but it was not optimized separately for each detector or mission. A deployment system could obtain a different precision–recall balance through calibration on a separate representative development dataset; the independent test missions themselves should not be used for such calibration.

### 4.4. Relation to Previous Sonar ATR Research

Direct numerical comparison with previously published sonar ATR results is not reliable because studies differ in sonar modality, target definitions, class composition, image dimensions, annotation completeness, data partitions, and evaluation metrics. Classification accuracy on pre-cropped contacts is particularly different from object-detection AP measured over complete missions containing large numbers of patches without annotated reference targets. The present study therefore does not claim superiority over results reported on other datasets.

Its main contribution relative to earlier comparisons is experimental scope. Previous work has compared CNN backbones for sonar classification [14], earlier detection algorithms [15], or multiple variants from the YOLO family [19]. Here, five detectors representing two-stage, dense one-stage, multi-scale one-stage, anchor-free, and transformer-based paradigms were evaluated using a common real-world development dataset and two complete held-out survey missions. All available test patches were processed, including those without annotated reference targets. This design exposes failure modes that can remain hidden when evaluation is restricted to cropped positive contacts or random image-level splits.

The mission-dependent ranking and the high false-alarm burden reinforce the recommendations in recent reviews for representative real-world datasets, standardized metrics, and mission-level evaluation [3,10,21]. They also indicate that incremental improvements measured within one sonar dataset should not be assumed to transfer unchanged to a new mission. Cross-mission verification should therefore accompany within-dataset validation whenever the intended use involves deployment beyond the acquisition conditions represented during model development.

### 4.5. Operational Implications

For AUV-based seabed surveys, the detector is only one component of a larger processing chain that includes sonar acquisition, image rendering, patch generation, navigation-data handling, contact aggregation, and operator review. The patch-level throughput measured in this study indicates that SSD, YOLOX, and DETR can process images faster than the two evaluated region/dense-prediction baselines on the test workstation. End-to-end latency on an AUV would additionally depend on the embedded processor, storage and data-transfer constraints, preprocessing cost, and the rate at which new sonar data are generated.

The patch-level FPS and memory values reported here were measured offline on a desktop-class GTX 1070 and should not be transferred directly to onboard AUV performance. Embedded computing platforms operate under substantially tighter computational, power, and thermal constraints, while end-to-end processing must additionally include sonar-record decoding, image rendering, patch generation, storage and data transfer, navigation-data handling, and contact aggregation. Consequently, even a detector that processes patches in real time on the desktop workstation may require additional optimization, such as reduced input resolution, quantization, pruning, or accelerator-specific compilation, to satisfy onboard latency and energy constraints. The present measurements should therefore be treated as a common-workstation baseline for selecting candidate configurations for later benchmarking on representative embedded AUV hardware.

The evaluation on complete, previously unseen real-world missions is particularly important from an operational perspective because it included target-containing patches and the much larger set of patches without an annotated reference target. The pronounced change in detector ranking between Test A and Test B, together with the substantial false-target burden, shows that favorable within-dataset validation does not translate directly into stable field performance. Marine ATR modules therefore remain sensitive to mission-specific domain shifts and still face substantial limitations in reliability, generalization, and false-alarm control before autonomous operational deployment can be considered.

Given the observed false-alarm burden, the evaluated models are presently better suited to decision support and contact prioritization than to fully autonomous final classification. A practical system could use detector confidence, temporal or spatial consistency across adjacent pings, acoustic shadow geometry, and navigation context to merge duplicate detections and rank contacts for operator inspection. Architectures combining high throughput with relatively few false target detections per patch without an annotated reference target, particularly YOLOX in the present tests, provide a useful basis for such a screening stage. However, mission-specific performance should be verified before operational use, and a human analyst should remain in the decision loop for safety-critical applications.

### 4.6. Limitations and Future Work

Several limitations define the scope of the conclusions. First, the development dataset was obtained from five missions and evaluation used two additional missions acquired with the same primary sonar model. This is a stronger test than a random patch split, but it does not represent the full range of sensors, frequencies, platforms, seabed types, geographic areas, and environmental conditions encountered in operational surveys. The study also evaluates generalization to new acoustic observations and mission contexts, not necessarily to previously unseen physical reference-object designs.

The development training–validation partition was constructed at patch level rather than by physical target, acoustic contact, or complete mission. Because repeated passes, channel-specific observations, and spatially shifted patches could correspond to the same physical reference contact, related observations may be represented in both development subsets. The validation results therefore characterize within-development model selection rather than an independent estimate of target- or mission-level generalization. The retrospective terminal-checkpoint sensitivity analysis reported here provides a limited post-hoc assessment of checkpoint dependence, but it does not remove the statistical dependence introduced by the patch-level development split and cannot substitute for grouped retraining. Moreover, because only one prespecified alternative checkpoint per configuration was evaluated, this analysis does not characterize the complete checkpoint trajectory. A future benchmark should therefore reserve a physical-target-, acoustic-contact-, or mission-grouped development subset for checkpoint selection before evaluation on final held-out missions.

Second, Test A and Test B differed markedly in their numbers of targets, and Test A contained only 21 annotated reference objects. Metrics from such a small target set can change substantially after a small number of detections or misses. Evaluation over a larger collection of complete missions is needed to estimate between-mission variability and confidence intervals more reliably.

Third, the non-target class was not exhaustively annotated in the independent missions. Test aggregates were therefore restricted to the two reference target classes, while the validation aggregate included three classes. This prevents direct interpretation of the validation-to-test difference as a single numerical generalization gap and limits the attribution of false alarms to specific benign-object categories.

Fourth, the annotations were prepared by two annotators who cross-reviewed one another’s work and corrected labels or bounding boxes when necessary. No formal inter-annotator agreement coefficient was calculated. Residual annotation uncertainty therefore cannot be quantified separately from model error.

Fifth, one training run was performed for each detector configuration, using an automatically generated and recorded seed. The results do not quantify variation across random initializations. Moreover, architecture-specific optimization and augmentation settings were retained, and a common maximum budget of 50 epochs may not correspond to the asymptotic optimum of every architecture. The reported comparison consequently concerns complete, resource-constrained configurations rather than isolated architectural effects. In particular, the configurations processed the same 200×200-pixel patch at model-input resolutions differing by more than an order of magnitude in pixel count, and the common 50-epoch limit is considerably shorter than the schedule under which the DETR architecture was originally characterized, as discussed in Section 2.7. Input resolution, augmentation strength, optimizer, and network topology are therefore mutually confounded, and no reported difference can be attributed to detection paradigm alone.

Sixth, environmental and operational variables were observed jointly rather than manipulated as independent factors. The performance differences between the missions cannot therefore be assigned causally to seabed type, water depth, vehicle altitude, target aspect, sonar range, or any other individual condition. Finally, inference speed was measured offline on a desktop GPU, and the study did not evaluate energy consumption, thermal limits, or end-to-end onboard processing latency.

Future work should extend the benchmark to additional complete missions, sonar systems, seabed conditions, and physical target designs. Exhaustive annotation of non-target contacts would support a more informative analysis of hard negatives and false-alarm sources. Repeated training with multiple seeds, mission-level resampling, and confidence intervals would allow the stability of model rankings to be quantified. A controlled comparison in which all configurations receive an identical model-input resolution would further separate the effect of detection paradigm from that of input scaling. Further work should also investigate hard-negative mining, confidence calibration on separate development missions, self-supervised sonar pretraining, domain adaptation, and the use of spatial context across adjacent patches or pings. For onboard deployment, pruning, quantization, and lightweight detector variants should be evaluated on representative embedded hardware. A final step should be an end-to-end sea trial in which detection, contact aggregation, georeferencing, and operator review are assessed as one operational pipeline.

## 5. Conclusions

This study compared Faster R-CNN, RetinaNet, SSD, YOLOX, and DETR for object detection in real AUV-acquired SSS imagery using a common development dataset and two independent mission tests. The within-development validation ranking served only for early stopping and checkpoint selection and is not interpreted as evidence of independent generalization. Faster R-CNN ranked first on the three-class validation subset, whereas SSD achieved the highest primary two-class ${\mathrm{mAP}}_{50:95}$ on Test A and YOLOX achieved the highest value on Test B. A retrospective comparison with the terminal training checkpoints showed that the Test A point estimates were more sensitive to checkpoint choice than those on Test B. Nevertheless, SSD remained the highest-performing configuration on Test A and YOLOX remained the highest-performing configuration on Test B under both checkpoint choices. These findings reinforce the need to base generalization conclusions on independent mission-level evaluation rather than on the within-development validation ranking. No detector configuration dominated all evaluation settings.

SSD achieved the highest observed accuracy on Test A with comparatively low training time, memory allocation, and inference cost. Among the evaluated configurations and missions, YOLOX showed a favorable combination of Test B accuracy, inference throughput, and false-reference-target alarm burden. These outcomes were mission dependent and, given the single training run per configuration and the configuration-specific input resolutions, augmentation policies, and optimization settings, should be interpreted as descriptive results of the complete evaluated configurations rather than as evidence for the superiority of any detector architecture or detection paradigm.

The principal contribution of this study is therefore methodological rather than architectural: it demonstrates the value of evaluating sonar detectors on complete held-out missions, including patches without annotated reference targets, and of reporting both confidence-ranked COCO metrics and threshold-dependent operating characteristics. Evaluation on previously unseen real-mission imagery demonstrated that favorable within-dataset validation does not guarantee stable field performance. The marked mission dependence and substantial false-target burden indicate that marine ATR modules still face major limitations in cross-mission generalization, reliability, and false-alarm control. The evaluated detectors may support contact screening and analyst assistance, but they do not yet justify fully autonomous safety-critical classification. Broader multi-mission evaluation, exhaustive hard-negative annotation, repeated training runs, domain-robust learning, confidence calibration, and embedded-system testing are required before operational deployment.

## Figures and Tables

**Table 1:** Characteristics of the AUV configurations used for acquisition of the sonar dataset.

Parameter	Gavia Defence AUV	LAUV
Vehicle class	Modular AUV	Lightweight modular AUV
Hull diameter	0.20 m	0.15 m
Approximate mass in air	70 kg; payload dependent	32 kg
Nominal depth rating of the study configuration	1000 m	100 m
Primary navigation sources	GPS, INS, DVL, pressure, and heading sensors	GPS, INS, DVL, pressure sensor, and acoustic-positioning support
Mission-control environment	Gavia Control Center	Neptus
Nominal commanded SSS survey speed	1.8 m/s	1.8 m/s
Primary imaging payload	EdgeTech 2205 SSS	EdgeTech 2205 SSS

**Table 2:** Nominal characteristics of the EdgeTech 2205 CHIRP channels used in the measurement campaigns, as specified by the manufacturer for the installed 600/1600 kHz side-scan configuration.

Parameter	LF CHIRP Channel	HF CHIRP Channel
Nominal center frequency	approximately 600 kHz	approximately 1600 kHz
Horizontal beamwidth	0.$33^{\circ }$	0.$20^{\circ }$
Vertical beamwidth at −3 dB	$60^{\circ }$	$60^{\circ }$
Nominal across-track resolution	15 mm	6 mm
Maximum ping rate	75 Hz	200 Hz
Peak source level	215 dB re 1 μPa	215 dB re 1 μPa

**Table 3:** Physical reference objects used during the measurement campaigns.

Detection Class	Nominal Geometry and Dimensions	Construction	Physical Exemplars
Manta-type	Low-profile body; base diameter 945 mm; height 440 mm	Welded steel structure	2
Cylindrical	Diameter 460 mm; length 1700 mm; wall thickness 12 mm	Welded steel structure	1

**Table 4:** Visualization profiles used during rendering of the development sonar records. TVG and normalization values denote processing-software settings rather than calibrated acoustic quantities.

Visualization Profile	TVG Setting	Normalization Factor
Bronze	100	0.10
Greenradar	80	0.09
Summer	80	0.09
Jet	120	0.20
Spring	120	0.25

**Table 5:** Candidate-patch quality-control flow before construction of the development dataset. The exclusion rate was calculated separately within each source group.

Source Group	Candidate Patches	Excluded	Exclusion Rate	Accepted Patches
Manta-associated	4464	648	14.52%	3816
Cylindrical-associated	2457	47	1.91%	2410
Non-target-associated	5862	22	0.38%	5840
**Total**	**12,783**	**717**	**5.61%**	**12,066**

**Table 6:** Class-wise distribution of annotated instances in the development dataset. Instance counts represent bounding-box annotations and not independent physical target exemplars.

Class	Training	Validation	Total
Manta-type objects	3019	759	3778
Cylindrical objects	1920	480	2400
Non-target seafloor objects	9748	2327	12,075
**Total**	**14,687**	**3566**	**18,253**

**Table 7:** Composition and purpose of the training and validation subsets.

Subset	Patches	Annotated Instances	Purpose
Training	9652	14,687	Model-parameter optimization
Validation	2414	3566	Early stopping, checkpoint selection, and within-dataset comparison
Development dataset	12,066	18,253	Common training and validation dataset

**Table 8:** Architectural families and concrete detector variants evaluated in this study. The comparison concerns the complete configurations listed in the table rather than the isolated influence of network topology.

Architecture	Evaluated Variant	Detection Paradigm	Principal Mechanism
Faster R-CNN	ResNet-50 with feature pyramid network	Two-stage detector	Region proposals followed by classification and bounding-box refinement
RetinaNet	ResNet-50 with feature pyramid network	Dense one-stage detector	Focal loss for foreground–background imbalance
SSD	SSD300 with VGG-based backbone	Multi-scale one-stage detector	Predictions from multiple feature-map resolutions
YOLOX	YOLOX-S	Anchor-free one-stage detector	Decoupled prediction head and dynamic label assignment
DETR	ResNet-50-based DETR	Transformer-based set prediction	Encoder–decoder attention and bipartite matching

**Table 9:** Input-resizing, batch, and augmentation settings used by the five detector configurations. The effective batch size was eight for every configuration.

Configuration	Model-Input Policy	Physical Batch	Accumulation	Training Augmentations
Faster R-CNN	Resize with aspect ratio preserved; configured scale up to 1333×800 pixels	4	2	Resize and random horizontal flip
RetinaNet	Resize with aspect ratio preserved; configured scale up to 1333×800 pixels	8	1	Resize and random horizontal flip
SSD	Fixed 300×300-pixel input	8	1	Expand, minimum-IoU random crop, photometric distortion, and random horizontal flip
YOLOX	Fixed 640×640-pixel input	8	1	Mosaic, random affine transformation, MixUp, and HSV-based photometric augmentation
DETR	Multi-scale random resizing; shorter dimension selected from 400–600 pixels and longer dimension limited to 1333 pixels	4	2	Random horizontal flip and multi-scale random-choice resizing

**Table 10:** Training convergence and computational cost for the five detector configurations. Peak memory denotes the maximum MMEngine memory statistic, corresponding to PyTorch max_memory_allocated. It represents allocator-reported tensor memory rather than a direct measurement of simultaneously resident dedicated VRAM.

Architecture	Best Epoch	Epochs Executed	Termination	Training Time	Peak MMEngine Memory
Faster R-CNN	15	25	Early stopping	15 h 10 min	6319 MB
RetinaNet	10	20	Early stopping	14 h 41 min	7134 MB
SSD	40	50	Epoch budget	7 h 11 min	5473 MB
YOLOX	47	50	Epoch budget	27 h 30 min	7031 MB
DETR	32	42	Early stopping	33 h 04 min	9509 MB

**Table 11:** Validation performance at each architecture’s selected checkpoint. The aggregate metrics include all three development classes.

Architecture	${\mathrm{mAP}}_{50:95}$	${\mathrm{mAP}}_{50}$	${\mathrm{mAP}}_{75}$	${\mathrm{AP}}_{S}$	${\mathrm{AP}}_{M}$	${\mathrm{AP}}_{L}$
Faster R-CNN	0.451	0.810	0.467	0.409	0.467	0.415
YOLOX	0.435	0.790	0.434	0.400	0.444	0.209
RetinaNet	0.429	0.785	0.431	0.393	0.442	0.492
SSD	0.400	0.759	0.381	0.361	0.420	0.386
DETR	0.391	0.761	0.349	0.340	0.432	0.414

**Table 12:** Recalculated two-class validation performance at the same selected checkpoints. Metrics are restricted to the Manta-type and cylindrical reference-target classes, providing a class-matched baseline for the independent mission tests.

Architecture	${\mathrm{mAP}}_{50:95}$	${\mathrm{mAP}}_{50}$	${\mathrm{mAP}}_{75}$	${\mathrm{AP}}_{S}$	${\mathrm{AP}}_{M}$	${\mathrm{AP}}_{L}$
Faster R-CNN	0.568	0.961	0.624	0.546	0.605	0.400
YOLOX	0.547	0.946	0.583	0.533	0.576	0.100
RetinaNet	0.537	0.930	0.570	0.524	0.571	0.510
SSD	0.508	0.922	0.508	0.491	0.552	0.375
DETR	0.497	0.923	0.466	0.454	0.569	0.400

**Table 13:** Composition of the two independent mission tests. Patches without an annotated reference target may still contain unannotated non-target seafloor objects.

Test	All Patches	Manta-Type	Cylindrical	All Targets	Patches Without Target Annotation
Test A	35,672	15	6	21	35,651
Test B	68,741	155	96	251	68,490

**Table 14:** COCO bounding-box performance on Test A and Test B. Aggregate values are averaged over the Manta-type and cylindrical classes only, with 95% bootstrap confidence intervals for the primary metric.

Test	Architecture	${\mathrm{mAP}}_{50:95}$ [95% CI]	${\mathrm{mAP}}_{50}$	${\mathrm{mAP}}_{75}$	${\mathrm{AR}}_{100}$	AP Manta-Type	AP Cylindrical
Test A	SSD	0.614 [0.529, 0.720]	0.869	0.791	0.737	0.470	0.758
Test A	Faster R-CNN	0.064 [0.022, 0.148]	0.166	0.056	0.492	0.100	0.028
Test A	RetinaNet	0.098 [0.043, 0.175]	0.178	0.081	0.692	0.106	0.090
Test A	YOLOX	0.289 [0.200, 0.445]	0.555	0.254	0.593	0.326	0.251
Test A	DETR	0.330 [0.184, 0.464]	0.566	0.370	0.637	0.180	0.480
Test B	SSD	0.065 [0.050, 0.090]	0.226	0.026	0.267	0.082	0.047
Test B	Faster R-CNN	0.071 [0.053, 0.096]	0.215	0.026	0.165	0.060	0.082
Test B	RetinaNet	0.033 [0.020, 0.058]	0.080	0.014	0.291	0.008	0.059
Test B	YOLOX	0.084 [0.068, 0.108]	0.314	0.011	0.266	0.111	0.056
Test B	DETR	0.060 [0.045, 0.084]	0.199	0.012	0.273	0.059	0.062

**Table 15:** Retrospective checkpoint-selection sensitivity on Test A and Test B. “Selected” denotes the checkpoint chosen exclusively by within-development validation, whereas “Terminal” denotes the final checkpoint reached by the original training run. Δ is Terminal minus Selected. Terminal-checkpoint results were not used for model selection.

Configuration	Selected Epoch	Terminal Epoch	Test A ${\mathrm{mAP}}_{50:95}$			Test B ${\mathrm{mAP}}_{50:95}$		
			Selected	Terminal	Δ	Selected	Terminal	Δ
SSD	40	50	0.614	0.643	+0.029	0.065	0.064	−0.001
Faster R-CNN	15	25	0.064	0.070	+0.006	0.071	0.069	−0.002
RetinaNet	10	20	0.098	0.189	+0.091	0.033	0.034	+0.001
YOLOX	47	50	0.289	0.299	+0.010	0.084	0.088	+0.004
DETR	32	42	0.330	0.256	−0.074	0.060	0.050	−0.010

**Table 16:** Target-class precision, recall, and F1-score at confidence 0.30 and IoU 0.50.

Test	Architecture	P Manta	R Manta	F1 Manta	P Cylindrical	R Cylindrical	F1 Cylindrical
Test A	SSD	0.205	1.000	0.341	0.023	1.000	0.045
Test A	Faster R-CNN	0.455	0.333	0.385	0.030	1.000	0.057
Test A	RetinaNet	0.093	0.267	0.138	0.022	1.000	0.042
Test A	YOLOX	0.160	1.000	0.275	0.025	1.000	0.049
Test A	DETR	0.012	1.000	0.024	0.014	1.000	0.027
Test B	SSD	0.333	0.542	0.413	0.034	0.562	0.064
Test B	Faster R-CNN	0.159	0.406	0.228	0.052	0.344	0.091
Test B	RetinaNet	0.033	0.148	0.054	0.023	0.260	0.043
Test B	YOLOX	0.344	0.652	0.450	0.046	0.448	0.084
Test B	DETR	0.034	0.594	0.064	0.033	0.396	0.061

**Table 17:** Inference throughput and false-target behavior on the independent mission tests.

Test	Architecture	Latency (ms/Patch)	FPS	False Detections/Patch	Total False Reference-Target Detections
Test A	SSD	30.72	32.55	0.257	9170
Test A	Faster R-CNN	95.35	10.49	0.760	27,078
Test A	RetinaNet	88.36	11.32	0.788	28,084
Test A	YOLOX	21.22	47.13	0.148	5275
Test A	DETR	53.46	18.71	0.947	33,773
Test B	SSD	31.80	31.45	0.460	31,510
Test B	Faster R-CNN	100.96	9.90	1.196	81,939
Test B	RetinaNet	91.62	10.91	1.013	69,364
Test B	YOLOX	20.26	49.35	0.253	17,305
Test B	DETR	53.74	18.61	1.467	100,496

## Data Availability

The raw sonar and navigation data are not publicly available due to institutional and operational restrictions. The processed data supporting the findings of this study may be made available by the corresponding author upon reasonable request, subject to approval by the Polish Naval Academy. To support reproducibility independently of access to the raw mission data, the Supplementary Materials provide the final detector configuration files, the exact COCO files defining the frozen training–validation partition, original training logs, evaluation and analysis scripts, recorded random-seed information, a de-identified dataset inventory, and the software and hardware environment record.

## Supplementary Materials

The following supporting information can be downloaded at: https://www.mdpi.com/article/10.3390/s26206486/s1, A Supplementary reproducibility package accompanies this article. It contains the final MMDetection configuration files for all five detector configurations, the original MMEngine training logs, the exact COCO annotation files defining the fixed training and validation subsets, the scripts used for independent-mission evaluation and checkpoint-sensitivity analysis, recorded random-seed information, a de-identified dataset inventory, and a software and hardware environment record. The package also contains the original DETR log entry underlying the reported maximum MMEngine memory value of 9509 MB.

## Abbreviations

The following abbreviations are used in this manuscript:

AP	Average precision
AR	Average recall
ATR	Automatic target recognition
AUV	Autonomous underwater vehicle
CI	Confidence interval
CNN	Convolutional neural network
COCO	Common Objects in Context
DETR	Detection Transformer
FPS	Frames per second
GPU	Graphics processing unit
IoU	Intersection over union
LAUV	Light Autonomous Underwater Vehicle
mAP	Mean average precision
PR	Precision–recall
R-CNN	Region-based Convolutional Neural Network
SSD	Single-Shot MultiBox Detector
SSS	Side-scan sonar
